# Mapping the literature on parents with mental illness, across psychiatric sub-disciplines: a bibliometric review

**DOI:** 10.1186/s12888-020-02825-4

**Published:** 2020-09-29

**Authors:** Njål Andersen, Ingunn Olea Lund

**Affiliations:** 1 Department of Leadership and Organizational Behaviour, BI Norwegian Business School, 0442 Oslo, Norway; 2 Department of Mental Disorders, The Norwegian Institute of Public Health, Postboks 222 Skøyen, 0213 Oslo, Norway; 3 Ludwig Boltzmann Gesellschaft, Research Group Village in cooperation with the Department of Psychiatry, Psychotherapy and Psychosomatics, Division of Psychiatry I, Medical University of Innsbruck, Tirol Kliniken GmbH, Schöpfstraße 23a, 6020 Innsbruck, Austria

**Keywords:** Bibliometric, Science mapping, Mental illness, Parent, Child

## Abstract

**Background:**

Research on parental mental illness is often carried out in disorder specific research silos. Drawing on the different research areas, it is possible to leverage and combine existing knowledge, and identify insights that can be transferred across research areas. In this study, we identify the overarching structure of research on parents with psychiatric disorders, and the structure of the different research areas, as defined by psychiatric disorder groups in ICD-10, and identify both topics that are commonly examined, and topics that received attention in only a few of the research areas.

**Methods:**

We use bibliometric science mapping to examine keywords in 16,734 articles, showing the overarching structure of research on parents with mental illness, both overall and within ICD-10 psychiatric disorder categories. The search was conducted using the Scopus database for journal articles published between 1999 and 2018, with no restrictions on language.

**Results:**

Co-occurrence analysis of the keywords in the 16,734 articles on parental mental illnesses in different psychiatric disorder categories, indicate there are six general themes in the literature: ‘expectant mothers and early motherhood’, ‘substance use and abuse’, ‘Socio-economic status’ (SES) and support practices’, ‘biomedical research‘, ‘diagnoses, symptoms and treatment’, and ‘child–parent interaction and context’. Although the same themes are covered in different areas, the contexts, in terms of content and relation to other topics, vary between the research areas. Some topics are heavily researched in some areas, but seem to be neglected in others.

**Conclusions:**

This study provides data both in interactive maps and an extensive table, allowing readers to dive deep into their topic of interest, and examine how this connects to other topics, which may in turn guide identification of important gaps in the literature, and ultimately inspire and generate novel research avenues.

## Background

There is a rapid increase in both the volume of research and topic specialization, where many scientists tend to focus on a narrow discipline. However, with the volumes of new research there are also ample opportunities for learning across the disciplines. A central challenge is to know what others, outside one’s own field are examining. In this report, we examine a topic in the field of psychiatry as a specific example.

Many children have a parent with a mental illness, and as a result are more likely to experience negative long-term adversities [1–5]. While there are well-established disciplines focusing on parents with different mental illnesses, the past decade has seen an increase in scholarship focusing on the children of parents with a mental illness (COPMI) [6–8]. Drawing on the different areas of research on parents with mental illnesses, it is possible to leverage and combine existing knowledge from these areas, and to identify insights that can be transferred and extended in the COPMI area. A review based on quantitative analysis of bibliographic data is well suited to realizing these opportunities and to providing a structural overview of the published research.

Although the term COPMI is relatively new, there is a wealth of literature on parents with mental illnesses for COPMI researchers to draw on, wherein the investigated outcomes range from factors related to the parents to those of the children. Research areas include parents with mood and affective disorders, anxiety disorders, psychotic disorders, personality disorders, behavioural syndromes, and substance use disorders, as defined in ICD-10’s chapter on mental disorders. The contribution of much of this research has not necessarily been to COPMI per se, but when examined in this context, it can yield valuable insights to COPMI research. This review will also illustrate to what extent there is an overlap in investigated topics across the aforementioned ICD-10 groups.

Science mapping, based on bibliometric analysis, offers a quantitative approach to analysing a large body of work and can be used to identify the central research topics and intellectual structures in a research area. In the proposed review, we intend to: first identify the overarching structure of research on parents with mental illnesses; identify the structure of each of the different research areas; and identify both topics that are commonly examined, and topics that received attention in only a few of the research areas. This shows how research topics fit together and provides an overview that cannot be obtained from other forms of reviews. Next, we present the data both in interactive maps and in an extensive table, allowing readers to dive deep into their topic of interest, and examine how this connects to other topics, which may in turn spark new ideas. Finally, by examining a few specific examples, we demonstrate the value and potential of exploring a topic through the science maps provided.

The proposed review aims to combine and synthesize existing COPMI literature with that on parents with mental illnesses and to identify topics where the latter can inform and move forward COPMI research. The results will allow COPMI scholars to see what research topics are more and less closely linked by enabling reviews of the diverse topics outside of their specific research areas and by facilitating information transfer from research outside of their own speciality. The results can help clinicians and researchers alike, researchers can obtain an overview of the key thematic area(s), guide identification of important gaps in the literature, and ultimately inspire and generate novel research avenues; and clinicians can get new insight about clinical problems they are struggling with due to limited research literature on a given topic from insights gained from a related field.

## Methods

### Search and inclusion criteria

We developed search terms based on each of the following six ICD-10 groups of mental illnesses: F10 - F19, substance use disorders (nicotine dependence was not included; F20-F29, Schizophrenia, schizotypal, delusional, and other non-mood psychotic disorders; F30-F39, mood and affective disorders; F40–48, anxiety, dissociative, stress-related, somatoform and other nonpsychotic mental disorders; F50-F59, behavioral syndromes associated with physiological disturbances and physical factors; and F60-F69, disorders of adult personality and behavior. To include articles that refer to generic terms, rather than only specific disorders, we also included a ‘generic psychiatric disorders’ search term. Each of the search terms are bounded by parental terms, selected to focus results on how parental mental illness can affect the children, instead of the other way around (see Additional file 1 table for a complete overview of the search terms). We examined a random sample of the resulting corpus to estimate proportion of articles that fall outside the subject, and estimate the rate at 7%, which is acceptable, as any distortions are likely washed out by constraints in the analysis. The search was conducted in April and August 2019, using the Scopus database for journal articles published between 1999 and 2018, with no restrictions on language. We selected the 20 year period starting from 1998, as the bibliographic data available in the database for earlier years are incomplete [9]. We selected the Scopus database for its wide reach, including all journals indexed by PubMed [10], and the full bibliographic data on published articles, which are unavailable in most other databases. Although the resulting corpus of articles may not be complete, we compared the results with searches in the Web of Science database, and the results indicate our corpus represents a sample of above the 85% of the total population of published journal articles recommended when using the network analysis [11] employed in this study.

**Table 1:** Overview of major topics in the themes in the overall research on ‘parental mental illness’, in articles published from 1999 to 2018; and across the research areas

		Research area/ disorder group						
Theme	Complete search	Substance use	Psychotic	Mood	Anxiety	Personality	Behavioral syndromes	Generic Psychiatric
Expectant mothers and early motherhood	Prescription drugs use during pregnancy; fetal monitoring, maternal and neonatal care; birth outcomes; eating and weight; sleep problems	Pregnancy and pregnancy complications, fetal monitoring, maternal and neonatal outcomes, opioid dependence, opioid maintenance treatment, neonatal abstinence syndrome; health services, health care delivery, health programs treatment	Prescription drugs, pregnancy complications, birth outcomes, birth parameters, prenatal, postnatal and maternal care, comorbidity	Prescription drug use during pregnancy, pregnancy complications, prenatal exposure, fetal monitoring; birth, maternal and neonatal outcomes, treatment	Prescription drug use during pregnancy, pregnancy complications, prenatal exposure, birth, maternal and neonatal outcomes, maternal diseases	*Covered in the theme ‘diagnoses, symptoms and treatment’	Pregnancy, complications, birth outcomes, birth parameters, postpartum mental health, SES, maternal care, neonatal care, pregnancy risk factorssocial support	Pregnancy, treatment, prescription drugs, prenatal exposure, birth outcomes, birth parameters, severe child outcome, maternal mental health, risk factors in pregnancy, demographics, SES, social support
Substance use and abuse	*Covered in the theme ‘SES, demography and health services’	Tobacco smoking, cannabis and marijuana, drug use and abuse; race and ethnicity	* Covered in the theme ‘diagnoses, symptoms and treatment’	*Covered in the theme ‘Child–parent interaction and context’	*Covered in the theme ‘Diagnoses, symptoms and treatment’	*Covered in the themes ‘diagnosis, symptoms and treatment’ and ‘animal and genetics’	*Covered in diagnosis theme: ‘other mental disorders’	Substance use and abuse, risk factors, SES, mental disorders, comorbidity
SES and support practices	Socioeconomic, demographic, and cultural factors; substance use and abuse; violence and crime		*SES covered in ‘diagnoses, symptoms and treatment’	Socioeconomic, demographic, cultural and ethnic factors, social support, health services and alcohol consumption	Socioeconomic, demographic, ethnic and cultural factors; health services, health behavior and counseling; social support	* Covered in themes ‘animal and genetics’ and ‘diagnosis, symptoms and treatment’	*SES occurs in the theme ‘expectant mothers and early motherhood’	*SES covered in ‘diagnoses, symptoms and treatment’
Biomedical research	Genetics, environment, gene-environment, animals, prenatal exposure, stress drugs, brain, brain development, cognition,	In utero exposure, cocaine and cocaine dependence, animals, brain development; genetic and environmental factors	**Two distinct themes** 1. animal and brain studies: animal, brain, pregnancy, prenatal exposure, environmental factor, cognitive outcomes	Animal and stress; genetic and environmental factors; brain and cognitive development and disorders	Animals, stress, maternal deprivation; brain and cognition; genetic and environmental factors and disease predisposition; prenatal exposure	Psychopathy, brain, genetics, families, SES, problem drinking, prenatal exposure, risk factor	Animals, brain, genetics, environment, treatment, health care, prescription drugs, symptoms,	Genetics, animals, brain, prenatal drug exposure, mental disorders, environmental factors
			2. genetics genetic risk, heredity, gene-environment interaction					
Diagnoses, symptoms and treatment	Health care -services, access, utilization, policies, programs and quality; treatment, social support, quality of life	*Treatment mainly covered in expectant mothers and early motherhood; and diagnoses and symptoms in child-parent interaction and context	Mental disorders, comorbidity, social support, treatment, child parent relation, substance use and abuse, SES, demography	Development disorders, psychotic disorders, hospitalization	Various mental disorders, comorbidity, substance use disorders, violence, including child abuse	Mental disorders, treatment, health services, comorbidity, substance abuse, pregnancy and pregnancy complications, violence	**Three distinct themes** 1. eating disorders, parental behavior, health information, child parent relation, families, lifestyle, coping	Mental disease, treatment, health care, child parent relation, behavior, SES, demography, social support
							2. Sleep disorders, behavior, learning, genetics, cognitive outcomes, health	
							3. Other mental disorders, comorbidity, substance abuse, violence	
Child–parent interaction and context	Child parent relation, various parental and child behaviors, attachment and adjustment, mental disorders, comorbidity, family functioning and social contexts	Alcohol use and abuse, drinking behavior; mental disorders, comorbidity, violence; parenting, child parent relation, behaviors, demography, SES, social support	*Covered in the theme ‘diagnoses, symptoms and treatment’	Child parent relation, various parental and child behaviors, including externalizing behaviors comorbidity, substance use and abuse, violence, suicide, family history	Child-parent relation, family functioning and communication, various phobia and anxiety, various treatments and therapies, externalizing behavior and disorders, autism spectrum disorders	Child parent relation, families, behaviors, personality, child development, violence, social support	*Covered in the sub-theme ‘eating disorders’ under ‘diagnoses, symptoms and treatment’	*Covered in the theme ‘diagnoses, symptoms and treatment’

### Data analysis

In this study, we employ bibliometric co-occurrence analysis on author and indexed keywords to identify how frequently topics are examined in the same article, and how keywords relate to each other in the wider context of research areas [12]. We conducted the analysis for each of the seven research areas indicated above, and on the combined areas, using the VOSviewer 1.6.12 software [13, 14]. The results enabled us to construct and understand a conceptual network structure of each of the areas and on an overarching perspective [15].

Prior to analysis, possible keywords were cleaned for generic terms such as the word ‘model’, ambiguous terms such as ‘time’, and words that do not denote a concept, such as names of countries and research methods. Finally, plural words were converted into their singulars, and synonyms were combined into a term. The principles for cleaning were devised by both authors based on past studies [16] and on independent coding of a 15% sample of all keywords. One author (IOL) constructed the thesaurus, which was subsequently verified by the other author (NA).

**Fig. 1:**
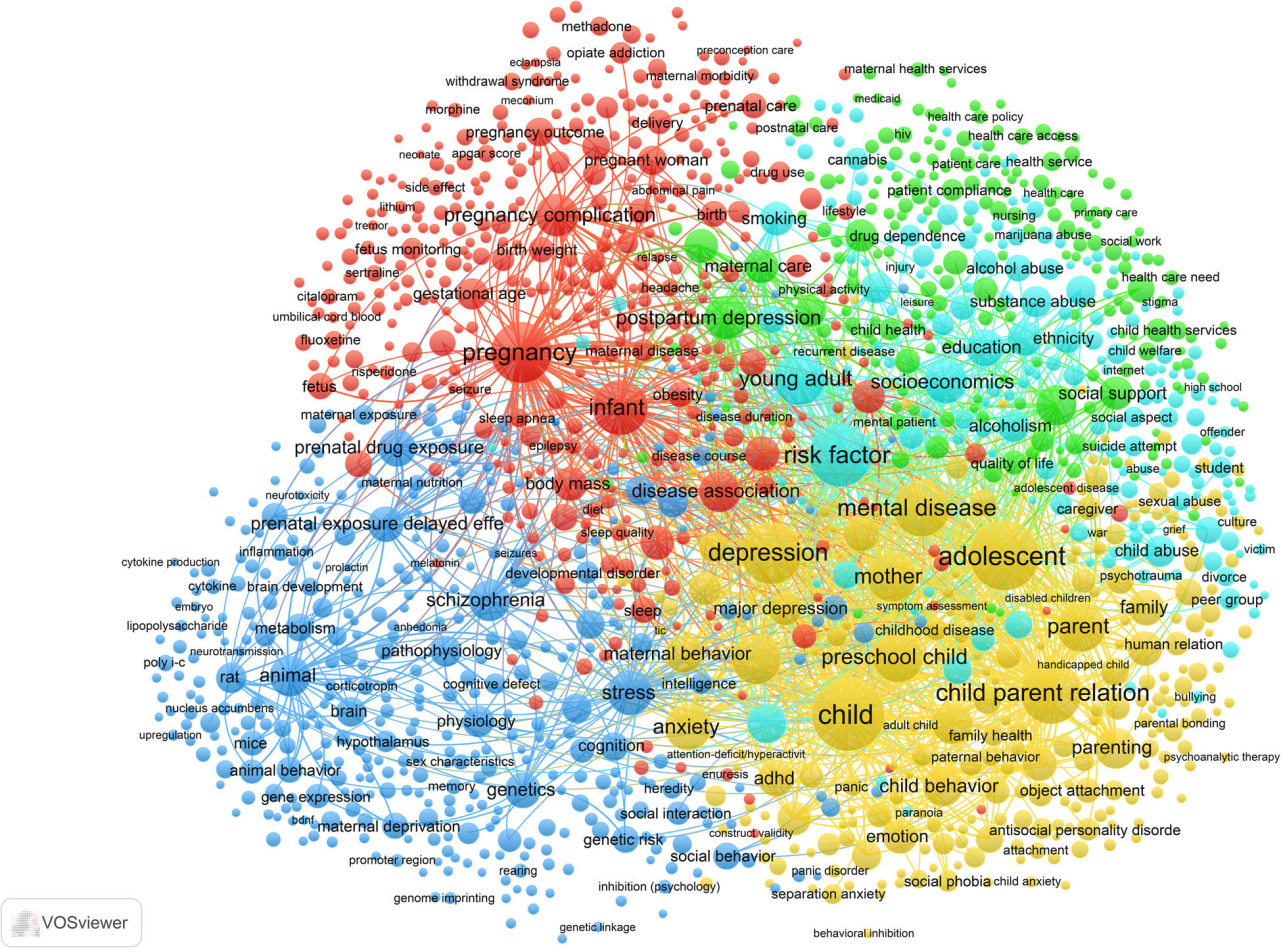
Keyword Co-Occurrence Network Graph for the Overall Research Field on Parental Mental Illness in the Period from 1999 to 2018. The graph is based on keywords in articles from the following research areas: ‘substance use disorders’; ‘psychotic disorders’; ‘mood and affective disorders’; ‘anxiety disorders’; ‘behavioral syndromes’; personality disorders’; and ‘generic psychiatric disorders’. Red cluster: Expectant mothers and early motherhood; Green cluster: Diagnoses, symptoms and treatment; Yellow cluster: Child–parent interaction and context; Turquoise cluster: SES and support practices; and Blue cluster: Biomedical research. Size of circle shows the relative number of occurrences of a keyword, and weight of line indicates the frequency two keywords are linked. Please note the number of lines in the figure has been reduced for legibility in the print edition. To view all the links, please access the interactive map using the Map and Network files found at OSF: https://osf.io/9ruva and the VOSviewer software

**Fig. 2:**
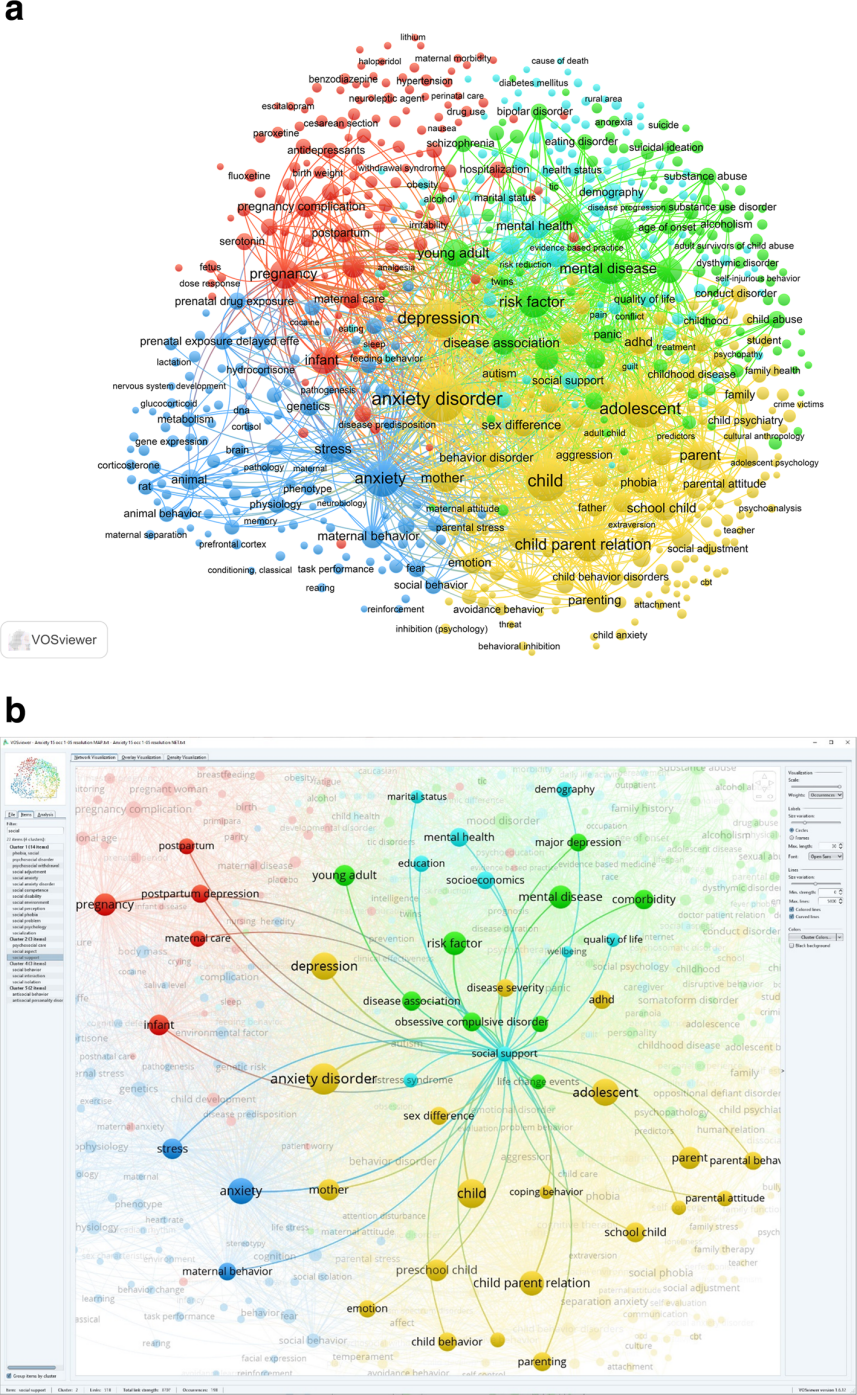
**a**. Keyword Co-Occurrence Network Graph for ‘Anxiety Disorders’ Area of Research, Based on Articles Published from 1999 to 2018. Red cluster: Expectant mothers and early motherhood; Green cluster: Diagnoses, symptoms and treatment; Yellow cluster: Child–parent interaction and context; Turquoise cluster: SES and support practices; and Blue cluster: Biomedical research. Size of circle shows the relative number of occurrences of a keyword, and weight of line indicates the frequency two keywords are linked. Please note the number of lines in the figure has been reduced for legibility in the print edition. To view all the links, please access the interactive map using the Map and Network files found at OSF: https://osf.io/9ruva and the VOSviewer software. Please see the appendix for maps of the other research areas and associated links to the interactive maps. **b**. A Section of Fig. 2a, Keyword Co-Occurrence Network Graph For the ‘Anxiety Disorders’ Area. This is what a section of the graph looks like when displayed in the interactive tool when ‘social support’ is selected to show other major keywords it is researched together with. The figure also show that in addition to linking with keywords within its own cluster, SES and support practices, the keyword ‘social support’ links with topics in all the other the clusters in the ‘anxiety disorders’ area

The bibliometric co-occurrence analysis was conducted on each of the seven research areas, with a lower limit set to 15 occurrences for each research area, and 30 for the combined search, meaning that only keywords appearing in 15 (or 30 for the combined search) or more articles are included in the analysis. The analysis yields a score for occurrence, a list of all keywords that occur together, and how the keywords cluster. To make these results accessible, keyword occurrences are presented in a table, and how they relate to each other is presented in a two-dimensional network graph. In the graph, the distance between keywords indicates relatedness, that is, the more similar keywords are, the closer together they are on the map; occurrence is reflected in the size of the circles, and the thickness of the lines indicates the number of articles the two co-occur in. Keywords that co-occur often are assigned to the same cluster. The layout and associated statistics were determined by a framework for mapping and clustering that is considered best practice [14], in the VOSviewer software [13]. The maps provide an overview of the overarching research field and the sub-domains, thus illustrating how the location of topics in relation to other topics can give an indication of possible gaps in the literature. Due to the richness of the data, we provide keyword co-occurrence maps for each of the research areas in the Additional file 1. These can be downloaded, searched, and explored interactively (see Figs. 1 and 2 as examples).

Some keywords connect the whole or parts of the network. These are termed ‘bridging keywords’ and due to their high centrality and associated relevance for a wide variety of keywords, the allocation to a given cluster is incidental. To identify keywords that act as bridges between major topics, we used the Pajek 5.05 software to calculate the betweenness centrality, a measure of how frequently a keyword is on the shortest path between other keywords [17].

To name the identified clusters based on prevalent themes, we followed the coding principles of grounded theory [18], where both authors independently undertook the steps of open and axial coding, to find common topics in the clusters. To reach a consensus, we conducted the selective coding process together and identified the final theme names (please see the Additional file 1 for more on the coding steps). The process included examining a more fine-grained cluster analysis of each co-occurrence map, and the use of network centrality measures to identify the most prominent terms.

Definitions: We use the term ‘area’ to describe an ICD-10 category, ‘themes’ to describe the general content clusters, ‘topics’ to describe individual parts of a theme, and ‘keyword’ to describe a specific concept, as used in articles.

## Results

### Identification of literatures

The combined search returned 16,734 articles, and for each area, returned: 2257 articles for ‘substance use disorders‘; 2568 for ‘psychotic disorders’; 3938 for ‘mood and affective disorders’; 3602 for ‘anxiety disorders’; 3861 for ‘behavioral syndromes’; 1166 for ‘adult personality and behavioral disorders’; and 5082 for ‘generic psychiatric disorders’. There is overlap between the areas, which explains why the total is less than the sum of the individual searches. There is a 53% overlap between the articles in the ‘generic psychiatric disorders’ search and the other searches; and a 16% overlap between all the other groups in total. The overlap is not corrected, as the aim is to identify topics examined in each domain. An article that contributes to more than one domain is therefore counted in each associated domain.

### Introduction to how themes are covered in different areas

A co-occurrence and cluster analysis of the keywords in the 16,734 articles on parental mental illnesses in the seven research areas, as defined by ICD-10, and the ‘generic psychiatric disorder’ search, indicate there are six general themes in the literature.

Figure 1 provides an overall structure of the research, based on the combined search. The analysis of each of the individual research areas shows large variations in focus, content and structure. Examining the results, we identified a set of themes that is prevalent to a greater or lesser extent in the different research areas. These are: ‘expectant mothers and early motherhood’, ‘substance use and abuse’, ‘SES and support practices’, ‘biomedical research’, ‘diagnoses, symptoms and treatment’, and ‘child–parent interaction and context’. Some themes such as ‘diagnoses, symptoms and treatment’ are covered in most of the research areas, while others, such as ‘substance use’, is only a distinct theme in some areas. Further, although the same theme is covered in different areas, the contexts, in terms of content and relation to other topics, tend to vary between the research areas. See Fig. 2, the keyword co-occurrence map for ‘mood’ disorder area, as an example.

In the following sections, we first outline the main themes, the central topics in each (see Table 1 for an overview), and how they relate to other topics and themes in the various research areas. Next, we provide a short description of the major bridging keywords. Finally, we present examples of keywords that are examined to varying extents in the different research areas, indicating both potential for cross areas learning, and identification of possible gaps in the literatures. A table of the 1408 keywords in Fig. 1 is available in the Additional file 1.

#### Theme 1: expectant mothers and early motherhood

The theme, ‘expectant mothers and early motherhood’, is distinct in all research areas apart from ‘personality’, and includes topics relating to the period from conception to the early period after birth, including pregnancy complications and related challenges. Other topics include: follow-up during pregnancy, description of birth, maternal and neonatal outcomes; and risk factors, such as substance use, and the role of health services and treatment of pregnant women, mothers and young children.

The role of prenatal exposure to legal and illegal substances is central in all areas. Prescription drug use during pregnancy is a dominant focus also in the ‘psychotic’, ‘mood’, ‘anxiety’, ‘generic psychiatric disorders’ areas. For example, in the ‘mood’ and ‘anxiety’ disorder areas, research is conducted on the safety of prescription drugs, such as antidepressants or anxiolytics, during pregnancy [19]. Illegal substance use is a dominant focus in both the ‘substance use disorders’ and ‘generic psychiatric disorder’ area. For example, opioid dependence, and the associated use of both legal and illegal opioids during pregnancy, is often studied in conjunction with maternal outcomes and the developing fetus and birth outcomes [20]. Topics in this theme frequently co-occur with topics in the ‘animal and genetics’ theme, for example on the effect of prenatal exposure to cocaine on the developing fetus and on brain development in animals [21]. A similar co-occurrence frequency is found in the safety of prescription drug use during pregnancy [22].

We also find that the role of social support is part of this theme in the ‘behavior syndromes’ and ‘generic psychiatric disorders’ research areas, such as social support interventions aimed at improving child outcomes [23].

#### Theme 2: child–parent interaction and contextual factors

The central topics in the ‘Child–parent interaction and contextual factors’ theme include various types of parental and child behaviors and mental disorders, and how these can influence the relationship between parents and their children. The role of contextual factors that can manifest as both risk and protective factors, and the role of comorbidity and other risk factors that can cause poor family functioning, are also central in this theme.

The theme is also distinct in the ‘substance use’, ‘mood’, ‘anxiety’ and ‘personality’ disorder areas; and receives somewhat less attention in the ‘psychotic’, ‘behavior syndromes’ and ‘generic psychiatric disorders’ areas, where the topic is covered as part of the ‘diagnoses, symptoms and treatment’ theme.

The ways that various externalizing behaviors affect the relationship between parents and their children are central in the ‘substance use’, ‘moods’, ‘anxiety’, ‘personality’ and ‘generic psychiatric disorders’ areas; for instance, in the ‘personality’ area, this theme is often studied in conjunction with keywords such as ‘violence’, ‘antisocial behavior’ and ‘conduct disorder’, and how these factors influence child outcomes [24, 25].

The role substance use and abuse has on the child–parent relation is prominent in the areas of ‘substance use’ and ‘personality disorders’. For example, keywords such as ‘, ‘substance abuse’, ‘alcohol abuse’, ‘cannabis addiction’, ‘opiate addiction’ and ‘cocaine-related disorders’, are examples of exposure variables related to outcomes such as family functioning [26–28].

Protective factors are frequently examined within this theme, in the ‘substance use’, ‘personality’, ‘behavior syndromes’ and ‘generic psychiatric disorders’ areas. One such protective factor is social support, which can act as a buffer against the influence of difficult family situations and upbringing and reduce stress and tensions in the family [23].

#### Theme 3: biomedical research

In the theme ‘biomedical research’ there are two distinct but connected topics; namely, research on animals, and on genetics. They are grouped as a theme in all the research areas.

There are two main foci within animal research: the first is on brain studies, the second on exposure to drugs. Brain research indicates a biological focus, with keywords such as dopamine, nerve cell, and prefrontal cortex. The second focus is on exposure, primarily on prenatal exposure to illegal and legal substances, including prescription drugs used to treat mental disorders. Animal studies are used to examine brain development and cognitive outcomes in the ‘psychotic’, ‘mood’, ‘anxiety’ and ‘generic psychiatric disorders’ area and often in relation to exposure variables, such as ‘cocaine’, ‘morphine’, ‘alcohol’ and dose-response of various types of substances in the ‘substance use’ area. Animal studies are often conducted to examine outcomes from exposure to various forms of stress in the ‘mood’, ‘anxiety’, ‘behavior’, ‘generic psychiatric disorders’, and ‘psychotic’ areas, with research on, for example, the associations between maternal stress during pregnancy and child emotional and cognitive problems [29], and on social behaviors in the ‘psychotic’ area [30].

The second focus in the theme is genetics. Keywords such as ‘twins’, ‘siblings’, and ‘gene-environment interaction’, are often studied in conjunction with intergenerational transmission of risk for mental disorders. For example, in the ‘personality’ area, genetics is often studied in conjunction with antisocial behavior and psychopathy, and in the ‘behavior syndromes’ area, sleep and eating disorders are heavily researched [31]. The genetic predisposition for developing alcohol and other substance use disorders is examined in the ‘substance use’ and ‘personality disorders’ areas [32]. The influence of genetic and environmental factors on child–parent relation is often studied in the ‘psychotic’ and ‘personality disorders’ areas [33].

Genetics research is also conducted using animal studies in the ‘substance use’, ‘psychotic’, ‘moods’, ‘anxiety’ and ‘generic psychiatric disorders’ areas. This includes research on the role of genetic animal models in developing and testing the safety of prescription drugs used for treating mental disorders [34, 35].

#### Theme 4: diagnoses, symptoms and treatment

The theme ‘Diagnoses, symptoms and treatment’ is a distinct theme in all but the ‘substance use disorders’ area. The central topics include various types of mental disorders and treatments, as well as topics related to health care. These topics are frequently related to comorbidity and various risk factors, such as substance use and abuse.

There are substantial variations across the areas, as to with which topics the theme is studied. Socioeconomic factors and demographic variables are common in the ‘psychotic’ and ‘generic psychiatric disorders’ areas, with research on, for example, how demographic factors are associated with higher prevalence of mental disorders. ‘Violence’ is a frequently associated topic in the ‘anxiety’, ‘personality’ and ‘behavioral syndromes’ areas, including keywords such as ‘domestic violence’, ‘child abuse’, ‘sexual abuse’, and ‘physical abuse’. For example, child abuse is examined as a risk factor for mental illness [36, 37] in the ‘personality’ area.

‘Social support’ is a prominent topic in the ‘psychotic’ area, with research on the extent social support provided to families with parental mental illness is associated with child behavior outcomes and child mental health and child–parent relationship [23].

In the ‘behavioral syndromes’ area, the theme is spread over three clusters. The first on eating disorders, often with a family focus [38]. The second on sleep disorders, often studied in conjunction with cognitive outcomes [39], and the third on ‘other mental disorders’, often studied in conjunction with comorbidity and externalizing problems [40].

#### Theme 5: substance use and abuse

Various types of substance use and substance use disorders make up the theme ‘substance use’, topics often studied in conjunction with socioeconomic and demographic factors, mental disorders, comorbidity, family relationships, violence and prenatal exposure.

As an independent theme, it is only present in two areas: ‘substance use disorders’ and ‘generic psychiatric disorders’, but it is present as topics in all of the areas. In both the ‘substance use disorders’ and the ‘generic psychiatric disorder’ areas, ‘substance use and abuse’ is often studied in conjunction with socioeconomic variables. For example identifying socioeconomic status, and financial strain as risk factors for substance use disorders [41], and violent communities as a risk factor for developing substance use disorders [42]. Further, research on the association between family environment, such as child abuse, and the risk of developing substance use and other mental disorders [37, 43] are common in the ‘generic psychiatric disorders’ area.

#### Theme 6: SES and support practices

The theme ‘SES, demography and health support’ is largely comprised of contextual topics, most often found as control variables or to frame a study, and while present in all areas, is only identified as an independent theme in two areas, namely ‘mood’ and ‘anxiety’ disorders. The theme has two main foci, the first relates to support practices, such as social support, community care and psychosocial care and various forms of support offered by professional health service providers. The second focus relates to the support programs, such as intended benefits, like prevention and risk reduction, and outcome measures, such as cost of illness and wellbeing.

In all the areas, SES and support practices are frequently studied with topics in the ‘child–parent interaction and context’ theme, specifically on topics such as education, social support and mental health. An example is how families in low SES neighborhoods experience more life stressors, and have less access to resources to deal with them [44]. In the ‘substance use’ and ‘psychotic disorders’ areas, SES is often included as both a risk factor, and control variable, for example, in conjunction with various mental disorders and treatments, [45] and with topics in the ‘expectant mothers and early motherhood’ theme, especially relating to post birth topics, such as postpartum depression [46].

### Bridging keywords

Some keywords are often used to describe a study setting, such as denoting a sample or general topic, and represent bridges between other keywords. We identified nine such keywords in this study (See Table 2). Five refer to sample characteristics in terms of child age groups, namely ‘infant’, ‘preschool child’, ‘child’, ‘school child’, ‘adolescent’ and ‘young adult’. Many studies take a risk perspective and the keyword ‘risk factor’ thus indicates such an approach. For example, parental age, SES and mental health are examined as risk factors for child maltreatment [47], and parental mental illness as a risk factor for offspring mental illness [48]. Similarly, ‘Mental disorders’ is a general and common term in the field; and ‘Depression’ is one of the most prevalent and debilitating health problems, often co-occurring with other mental health problems [49, 50] and extensively researched [51].

**Table 2:** Number occurrences of a selection of keywords in each of the research areas ^a^

	Combined Search	Substance Use Disorders	Behavioral syndromes	Adult personality and behavior	Psychotic disorders	Mood disorders	Anxiety disorders	Generic psychiatric disorder terms
Selection of keywords								
Social support	917	75	281	48	85	240	198	269
Outpatient care	150	25	40	..	21	42	34	62
Child abuse	825	156	91	141	79	245	173	325
Domestic violence	269	73	32	23	26	68	55	111
Intimate partner violence	140	35	32	..	..	33	30	44
Emotional abuse	93	15	..	..	..	28	22	41
Bridging words								
Risk factor	4593	740	1000	396	758	1329	938	1395
Depression	4431	254	824	290	442	2364	1616	1095
Mental disease	3641	266	354	302	623	914	845	2624
Child sample characteristics								
Infant	3321	530	1021	151	398	725	587	735
Preschool child	2651	189	756	174	173	626	682	835
School child	2651	189	756	174	173	626	682	835
Adolescent	6093	941	1165	543	771	1616	1485	1947
Young adult	2751	481	629	210	417	741	610	884
Child	6896	710	1335	533	866	1792	1862	2179

*Notes*. ^a^These are presented to illustrate the extent to which topics are covered overall in the combined search, and across different research areas. An occurrence corresponds to an article with the keyword. Please see the Additional file 1 for the complete table, with the 1408 keywords that have more than 30 occurrences in the combined search

### Interacting with the results

A main aim of this study is to enable researchers to explore the extent to which specific topics occur across the different areas, and how they co-occur with other topics. To do so, we provide a table with the occurrence across the different areas of the 1408 keywords with more than 30 occurrences in the combined search results in the Additional file 1. We have extracted example terms for Table 2, such as social support, which occurs in more than 900 articles. However, the amount of attention received by the topic varies across the areas, and it is most extensively researched in the ‘behavior syndromes’, ‘mood’ and the ‘generic psychiatric disorders’ areas. The term ‘child abuse’, occurs in more than 800 articles in the overall search, with substantially more research conducted in the ‘moods‘and ‘generic psychiatric disorder‘areas than in the ‘psychotic disorders’ area.

For researchers interested in particular stages of child development, Table 2 also illustrates substantial variation regarding which age groups most often constitutes the study sample in different areas. For instance, preschool children receive more attention in the mood, anxiety, behavior syndromes and generic psychiatric disorders areas, than in the other areas.

To identify what other keywords a given term is most frequently researched with, it is possible to locate the term in the interactive network maps, and highlight the associated terms, either for each individual research area, or for the field in total.

## Discussion

We identified the overarching structure of research on parents with mental illnesses, and identified similarities and differences in the structure of the different research areas. Our search returned the majority of results when using disorder-specific keywords, suggesting that most research focuses on a parent’s specific diagnosis rather than generic psychiatric disorders and that the literatures are separate. With the interactive network maps and the table of keyword occurrences across the research areas, this review is the first to identify topics commonly examined across areas and those primarily studied in a few areas. As such, the review shows how the research fits together and provides an overview that cannot be obtained from other types of reviews.

Based on co-occurrence analysis of keywords from more than 16,000 articles, we show that the literature consists of six major themes. There are clear differences in the different areas, awareness of which can help researchers identify and connect results from studies in areas outside of their own; and may spark new research ideas. This may be of particular value for emerging fields such as COPMI.

Comparing how the various themes are covered in individual research areas, we see substantial variation in both the number of articles and the topics they are studied in conjunction with. ‘Social support’ represents an example, where the topic has received significant attention in the ‘mood’, ‘behavioral syndromes’ and ‘generic psychiatric disorder’ (more than 200 articles), while it has received scant attention in the ‘substance use’ and ‘personality disorders’ areas. A second example is that ‘child abuse’, is studied most frequently in the ‘mood’ and ‘generic psychiatric disorders’ areas, and less so in the ‘psychotic disorders’ and ‘behavioral syndromes’ areas. These, and other similarly skewed topics, represent potentially valuable avenues of research. Social support can help most psychiatric patients and their family members; and knowledge is needed on how to best facilitate it in *all* the research areas on parental mental illness. As for child abuse, we can think of no good reasons why this is less of an issue in some research areas than in others; knowledge on how to prevent such cases is important across all research areas on parental mental illness.

Some research areas are more similar than others: ‘mood’ and ‘anxiety’ look similar both in terms of structure and topics. However, close examination reveals substantial variation in the topics that are most commonly examined and what topics are studied in conjunction with each other. For instance, social support is covered in both research areas. However, in the ‘moods’ area, it is often studied in conjunction with socioeconomics and pregnancy topics, which is not the case in the ‘anxiety’ research area.

These examples further illustrate that knowledge on a given topic may be dominated by research in a single, or few areas. Such skewness may represent a source of bias emanating from idiosyncrasies in a given area, not necessarily evident to a specialist researcher. The results presented in this review may offer an indication as to whether this is the case.

### Limitations

Several limitations should be mentioned. First, a bibliometric review can only provide an overview of a research area; thus it is no substitute for extensive reading, and should be seen as a complement, not a replacement of narrative, scoping or systematic reviews. More in-depth reviews are necessary when the aim is to identify specific mechanisms and evaluate the strength and quality of the research articles included in the review. Second, there are no quality measures of the journals and articles included, apart from requirements to be indexed by Scopus. Third, despite our best efforts, the data represents a sample, rather than all published articles on parents with a mental illness in the time period 1999 and 2018.

## Conclusion

The present study is the first review, to our knowledge, to map the structure of research on parents with mental illnesses as a whole; as well as similarities and differences in the structure and research focus in the various areas as defined by the ICD-10. The study can help both scholars and clinicians get an overview of the entire research field, and it can facilitate identifying important gaps in the literature, and promising new research avenues. Although we only presented a few examples, the results present a wealth of data, from which a range inferences and inspirations may be drawn. We hope to facilitate utilization of the results for these purposes by providing interactive and searchable network maps and a complete overview of keywords and their occurrence across the research areas, thus provoking thought in the reader and inspiration for scholars to identify, examine and explore novel and valuable ways to advance the area.

## Supplementary information

**Additional file 1.** Search terms, complete set of bibliometric network diagrams, and extended table comparing keyword occurrences in each of the research areas.

## Data Availability

Raw data can be accessed by using the search strings we made available in the Additional file 1, and repeating the search in Scopus. In addition, all the files needed to access the network graph files can be accessed through the project folder at the open science framework (OSF) site: https://osf.io/9ruva. These files can be opened in the freely available VOSviewer software.

## Abbreviations

COPMI: Children of parents with a mental illness
ICD-10: International classification of diseases, tenth revision
SES: Socio-economic status
