# Supplementary material for: Mapping the literature on parents with mental illness, across psychiatric sub-disciplines: a bibliometric review
Source: BMC Psychiatry. 2020 Sep 29;20:468. doi: 10.1186/s12888-020-02825-4 (PMC7523296; doi:10.1186/s12888-020-02825-4)
Supplement: Supplementary file 1 — Additional file 1. Search terms, complete set of bibliometric network diagrams, and extended table comparing keyword occurrences in each of the research areas. [file 12888_2020_2825_MOESM1_ESM.pdf]

Appendix:
Mapping the literature on parents with mental illness, across psychiatric sub-disciplines: A bibliometric review

Njål Andersen1 & Ingunn Olea Lund2,3*

1 Department of Leadership and Organizational Behaviour, BI Norwegian Business School, 0442 Oslo, Norway
2 The Norwegian Institute of Public Health, Postboks 222 Skøyen, 0213 Oslo, Norway
3Ludwig Boltzmann Gesellschaft, Research Group Village in cooperation with the Department of Psychiatry, Psychotherapy and Psychosomatics, Division of Psychiatry I, Medical University of Innsbruck, Tirol Kliniken GmbH, Schöpfstraße 23a, 6020 Innsbruck, Austria

Table of content

Search terms: 2
Keyword co-occurrence network diagrams of each of the research areas: 11
Complete table 2: Number of keyword occurrences in each of the research areas: 19

Search terms

The following search terms were used for each of the searchers.

Psychotic disorders:

PUBYEAR AFT			1998			
						AND
DOCTYPE	(		ar		)	
						AND
TITLE-ABS-KEY	(					
	(	“	parental	“	)	OR
	(	“	maternal	“	)	OR
	(	“	paternal	“	)	OR
	(	“	young carer*	“	)	
	)					
						AND
TITLE-ABS-KEY	(					
	(	“	Schizophren*	“	)	OR
	(	“	Schizotypal disorder*	“	)	OR
	(	“	Delusional disorder*	“	)	OR
	(	“	psychotic* disorder*	“	)	OR
	(	“	Schizoaffective disorder*	“	)	OR
	(	“	psychosis*	“	)	
	)					
						AND
PUBYEAR			< 2019			

Behavioral syndromes:

PUBYEAR AFT			1998			
						AND
DOCTYPE	(		ar		)	
						AND
TITLE-ABS-KEY	(					
	(	“	parental	“	)	OR
	(	“	maternal	“	)	OR
	(	“	paternal	“	)	OR
	(	“	young carer*	“	)	
	)					
						AND
TITLE-ABS-KEY	(					
	(	“	eating disorder*	“	)	OR
	(	“	anorexia*	“	)	OR
	(	“	bulimia*	“	)	OR
	(	“	sleep* disorder*	“	)	OR
	(	“	isonmia*	“	)	OR
	(	“	hypersomnia*	“	)	OR
	(	“	Postpartum depression	“	)	
	)					
						AND
PUBYEAR			< 2019			

Adult personality and behavior:

PUBYEAR AFT			1998			
						AND
DOCTYPE	(		ar		)	
						AND
TITLE-ABS-KEY	(					
	(	“	parental	“	)	OR
	(	“	maternal	“	)	OR
	(	“	paternal	“	)	OR
	(	“	young carer*	“	)	
	)					
						AND
TITLE-ABS-KEY	(					
	(	“	personality disorder*	“	)	OR
	(	“	impulse disorder*	“	)	OR
	(	“	gender identify disorder*	“	)	OR
	(	“	parahilias	“	)	OR
	(	“	sexual disorder*	“	)	OR
	(	“	disorder* of adult personality and behavior*	“	)	
	)					
						AND
PUBYEAR			< 2019			

Generic psychiatric disorders:

PUBYEAR AFT 1998
AND
DOCTYPE (ar)
AND
TITLE-ABS-KEY (
(“ parental “) OR
(“ maternal “) OR
(“ paternal “) OR
(“ young carer* “)
)
AND
TITLE-ABS-KEY (
(“ mental disorder* “) OR
(“ mental illness* “) OR
(“ psychiatr* disorder* “) OR
(“ psychiatr* illness* “)
)
AND
PUBYEAR < 2019

Substance use disorders:

PUBYEAR AFT 1998
AND
DOCTYPE (ar)
AND
TITLE-ABS-KEY (
(“ parental “) OR
(“ maternal “) OR
(“ paternal “) OR
(“ young carer* “)
)
AND
TITLE-ABS-KEY (
(“ Alcohol* abuse* “) OR
(“ Alcohol* depend* “) OR
(“ Alcohol use disorder* “) OR
(“ Alcohol* disorder* “) OR
(“ Alcohol* addict* “) OR
(“ opioid* disorder* “) OR
(“ opioid* abuse* “) OR
(“ opoid* dependen* “) OR
(“ opioid* use* “) OR
(“ opioid* addict* “) OR
(“ cannabis disorder* “) OR
(“ cannabis* abuse* “) OR
COPMI (“ cannabis* addict* “) OR
(“ cannabis* dependen* “) OR
(“ axiolytic* abuse* “) OR
(“ anxiolytic* dependen* “) OR
(“ anxiolitic* addict* “) OR
(“ anxiolytic* disorder* “) OR

(“ hypnotic* abuse* “) OR
(“ hypnotic*dependen* “) OR
(“ hypnotic* addict* “) OR
(“ hypnotic* disorder* “) OR
(“ sedative* abuse* “) OR
(“ sedative* dependen* “) OR
(“ sedative* addict* “) OR
(“ sedative* disorder* “) OR
(“ methamphetamine* abuse* “) OR
(“ methamphetamine* dependen* “) OR
(“ methamphetamine* addict* “) OR
(“ methamphetamine* disorder* “) OR
(“ amphetamine* abuse* “) OR
(“ amphetamine* dependen* “) OR
(“ amphetamine* addict* “) OR
(“ amphetamine* disorder* “) OR
(“ cocaine* disorder* “) OR
(“ cocaine* abuse* “) OR
(“ Cocaine* depend* “) OR
(“ Cocaine* use* “) OR
(“ stimulant* disorder* “) OR
(“ stimulant* abuse* “) OR
(“ stimulant* depend* “) OR
(“ stimulant* use “) OR
(“ stimulant* addict* “) OR
(“ cocaine* addict* “) OR
(“ Hallucinogen* disorder* “) OR
(“ Hallucinogen* abuse* “) OR
(“ Hallucinogen* depend* “) OR
(“ Hallucinogen* use “) OR
(“ Hallucinogen* addict* “) OR
(“ Inhalant* disorder* “) OR
(“ Inhalant* abuse* “) OR
(“ inhalant* depend* “) OR
(“ inhalant* addict* “) OR
(“ psychoactive substance* disorder* “) OR
(“ psychoactive substance* abuse* “) OR
(“ psychoactive substance* depend* “) OR
(“ psychoactive substance* addict* “) OR
(“ opiate* addict* “) OR
(“ opiate* disorder* “) OR
(“ opiate* depend* “) OR
(“ opiate* abuse* “) OR
(“ marijuana* addict* “) OR
(“ marijuana* disorder* “) OR
(“ marijuana* depend* “) OR
(“ marijuana* abuse* “)
)

AND

PUBYEAR < 2019

Anxiety disorders:

PUBYEAR AFT	1998	
		AND
DOCTYPE	(ar)	
		AND
TITLE-ABS-KEY	(	
	(“ parental “)	OR
	(“ maternal “)	OR
	(“ paternal “)	OR
	(“ young carer* “)	
	)	
		AND
TITLE-ABS-KEY	(	
	(“ anxiety disorder* “)	OR
	(“ Obsessive-compulsive disorder* “)	OR
	(“ Somatoform disorder* “)	OR
	(“ phobic* “)	OR
	(“ phobia “)	OR
	(“ Dissociative disorder* “)	
	)	
		AND
PUBYEAR	< 2019	

Mood disorders:

PUBYEAR AFT	1998	
		AND
DOCTYPE	(ar)	
		AND
TITLE-ABS-KEY	(	
	(“ parental “)	OR
	(“ maternal “)	OR
	(“ paternal “)	OR
	(“ young carer* “)	
	)	
		AND
TITLE-ABS-KEY	(	
	(“ Manic episode* “)	OR
	(“ Bipolar disorder* “)	OR
	(“ depressive disorder* “)	OR
	(“ mood* disorder* “)	OR
	(“ affective disorder* “)	
	)	
		AND
PUBYEAR	< 2019	

Combined search:

PUBYEAR AFT			1998			
						AND
DOCTYPE	(		ar		)	
						AND
TITLE-ABS-KEY	(					
	(	“	parental	“	)	OR
	(	“	maternal	“	)	OR
	(	“	paternal	“	)	OR
	(	“	young carer*	“	)	
	)					
						AND
TITLE-ABS-KEY	(					
	(	“	personality disorder*	“	)	OR
	(	“	impulse disorder*	“	)	OR
	(	“	gender identify disorder*	“	)	OR
	(	“	parahilias	“	)	OR
	(	“	sexual disorder*	“	)	OR
	(	“	disorder* of adult personality and behavior*	“	)	OR
	(	“	eating disorder*	“	)	OR
	(	“	anorexia*	“	)	OR
	(	“	bulimia*	“	)	OR
	(	“	sleep* disorder*	“	)	OR
	(	“	isonmia*	“	)	OR
	(	“	hypersomnia*	“	)	OR
	(	“	Postpartum depression	“	)	OR
	(	“	Manic episode*	“	)	OR
	(	“	Bipolar disorder*	“	)	OR
	(	“	depressive disorder*	“	)	OR
	(	“	mood* disorder*	“	)	OR
	(	“	affective disorder*	“	)	OR
	(	“	anxiety disorder*	“	)	OR
	(	“	Obsessive-compulsive disorder*	“	)	OR
	(	“	Somatoform disorder*	“	)	OR
	(	“	phobic*	“	)	OR
	(	“	phobia	“	)	OR
	(	“	Dissociative disorder*	“	)	OR
	(	“	Schizophren*	“	)	OR
	(	“	Schizotypal disorder*	“	)	OR
	(	“	Delusional disorder*	“	)	OR
	(	“	psychotic* disorder*	“	)	OR
	(	“	Schizoaffective disorder*	“	)	OR
	(	“	psychosis*	“	)	OR
	(	“	opioid* disorder*	“	)	OR
	(	“	opioid* abuse*	“	)	OR
	(	“	opoid* dependen*	“	)	OR
	(	“	opioid* use*	“	)	OR
	(	“	opioid* addict*	“	)	OR
	(	“	cannabis disorder*	“	)	OR
	(	“	cannabis* abuse*	“	)	OR
	(	“	cannabis* addict*	“	)	OR
	(	“	cannabis* dependen*	“	)	OR
	(	“	axiolytic* abuse*	“	)	OR

(“ anxiolytic* dependen* “) OR
(“ anxiolitic* addict* “) OR
(“ anxiolytic* disorder* “) OR
(“ hypnotic* abuse* “) OR
(“ hypnotic*dependen* “) OR
(“ hypnotic* addict* “) OR
(“ hypnotic* disorder* “) OR
(“ sedative* abuse* “) OR
(“ sedative* dependen* “) OR
(“ sedative* addict* “) OR
(“ sedative* disorder* “) OR
(“ methamphetamine* abuse* “) OR
(“ methamphetamine* dependen* “) OR
(“ methamphetamine* addict* “) OR
(“ methamphetamine* disorder* “) OR
(“ amphetamine* abuse* “) OR
(“ amphetamine* dependen* “) OR
(“ amphetamine* addict* “) OR
(“ amphetamine* disorder* “) OR
(“ cocaine* disorder* “) OR
(“ cocaine* abuse* “) OR
(“ Cocaine* depend* “) OR
(“ Cocaine* use* “) OR
(“ stimulant* disorder* “) OR
(“ stimulant* abuse* “) OR
(“ stimulant* depend* “) OR
(“ stimulant* use “) OR
(“ stimulant* addict* “) OR
(“ cocaine* addict* “) OR
(“ Hallucinogen* disorder* “) OR
(“ Hallucinogen* abuse* “) OR
(“ Hallucinogen* depend* “) OR
(“ Hallucinogen* use “) OR
(“ Hallucinogen* addict* “) OR
(“ Inhalant* disorder* “) OR
(“ Inhalant* abuse* “) OR
(“ inhalant* depend* “) OR
(“ inhalant* addict* “) OR
(“ psychoactive substance* disorder* “) OR
(“ psychoactive substance* abuse* “) OR
(“ psychoactive substance* depend* “) OR
(“ psychoactive substance* addict* “) OR
(“ Alcohol* abuse* “) OR
(“ Alcohol* depend* “) OR
(“ Alcohol use disorder* “) OR
(“ Alcohol* disorder* “) OR
(“ Alcohol* addict* “) OR
(“ opiate* addict* “) OR
(“ opiate* disorder* “) OR
(“ opiate* depend* “) OR
(“ opiate* abuse* “) OR
(“ marijuana* addict* “) OR
(“ marijuana* disorder* “) OR

(“ marijuana* depend* “) OR
(“ marijuana* abuse* “) OR
(“ mental disorder* “) OR
(“ mental illness* “) OR
(“ psychiatr* disorder* “) OR
(“ psychiatr* illness* “)
)
AND
PUBYEAR < 2019

Keyword co-occurrence network diagrams of each of the research areas

Static visualization of the keyword co-occurrence network maps, which were created using VOSviewer 1.6.12 software. Only keywords with more than 30 instances, meaning there are more than 30 articles with the given keyword, are included in the 'Complete search' map. Similarly, the cutoff for the other maps is set at 15. The size of circle shows relative number of occurrences of a keyword, weight of line indicates the frequency of keywords are linked. Only the 1000 most frequent links are shown for clarity.

To examine how individual keywords interlink, and adjust the resolution (to get a higher degree of clustering), please access the searchable and interactive version of the maps. To access the individual maps, please visit the Open Science Foundation (OSF) data repository **https://osf.io/9ruva/**, download the files and open them in VOSviewer. For instructions on how to navigate the map, please see the VOSViewer manual: http://www.vosviewer.com/getting-started#VOSviewer

Complete search

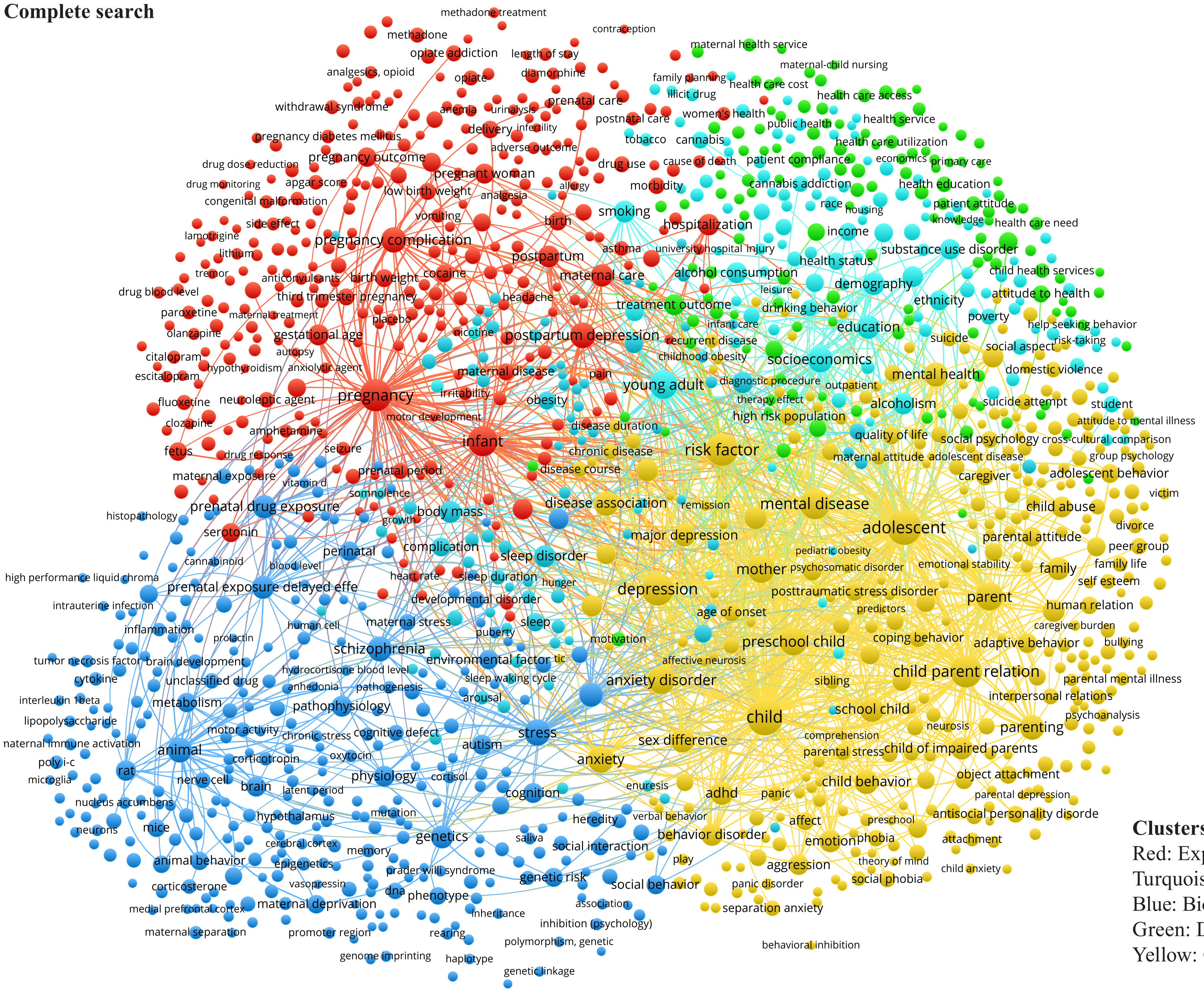

Clusters:
Red: Expectant mothers and early motherhood
Turquoise: SES and support practices
Blue: Biomedical research
Green: Diagnoses, symptoms and treatment
Yellow: Child – parent interaction and context

Behavioral syndromes

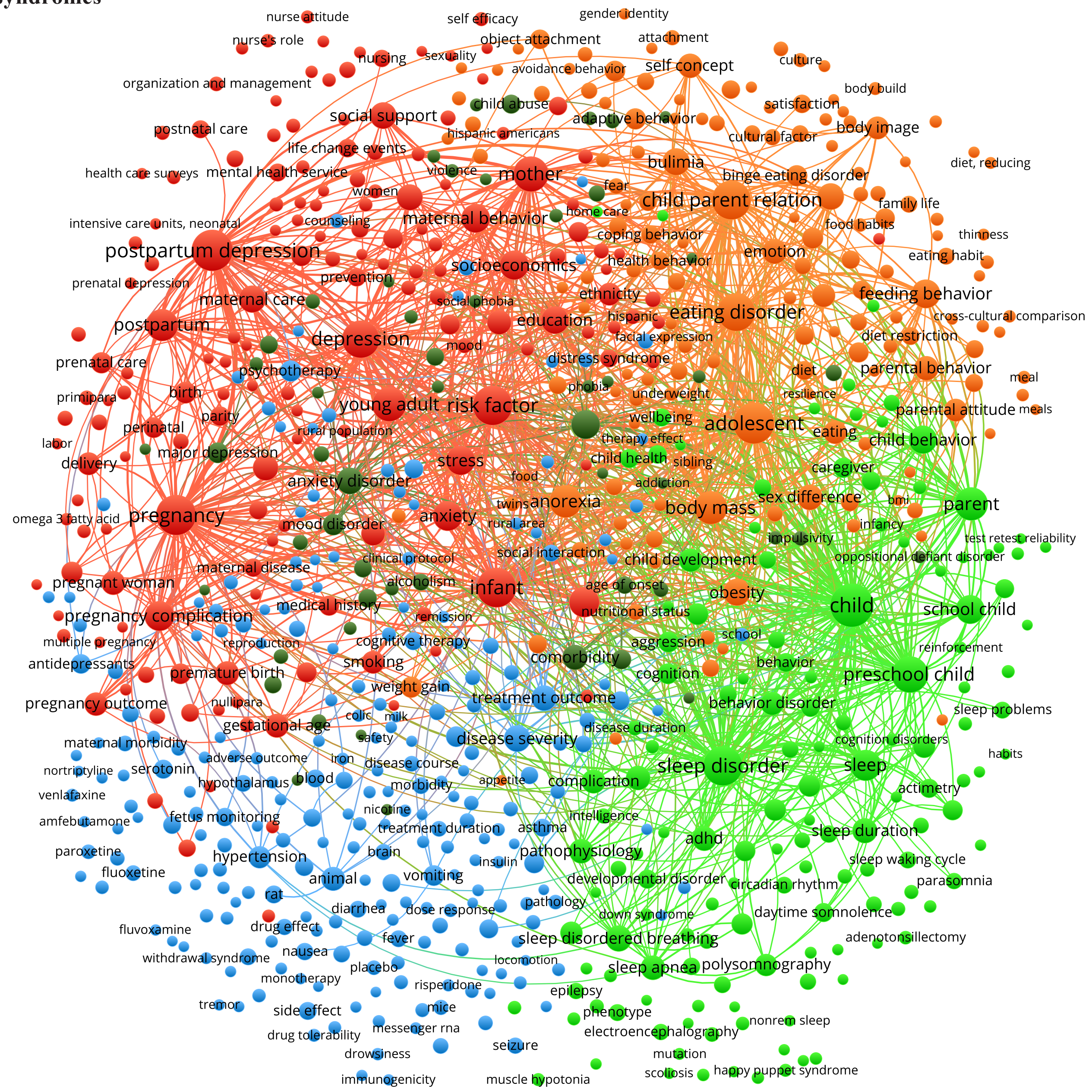

Clusters:
Red: Expectant mothers and early motherhood
Blue: Biomedical research
Green: Diagnoses, symptoms and treatment
Dark Green: Other mental disorders
Orange: Child – parent interaction and context

Anxiety disorder

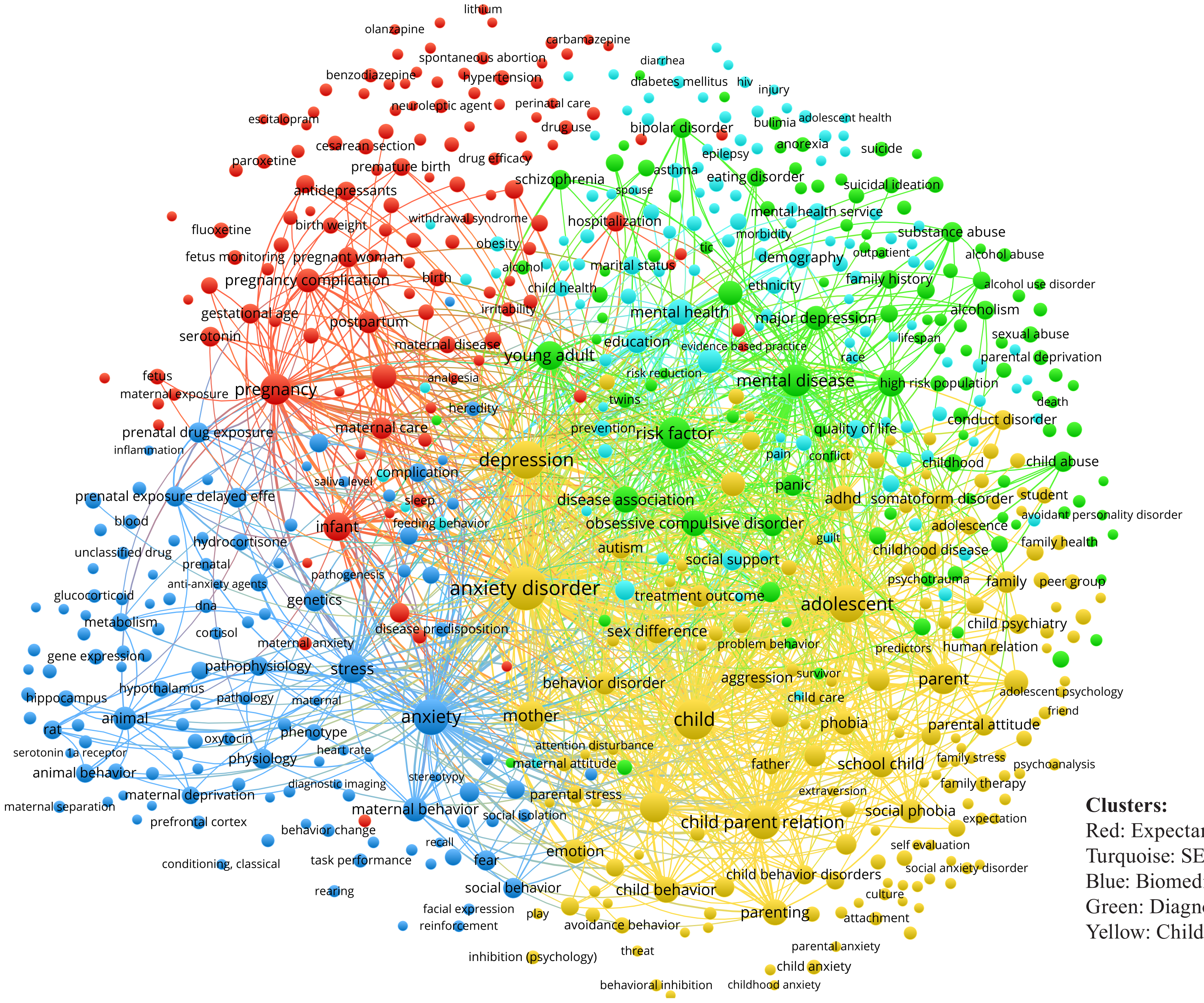

Clusters:
Red: Expectant mothers and early motherhood
Turquoise: SES and support practices
Blue: Biomedical research
Green: Diagnoses, symptoms and treatment
Yellow: Child – parent interaction and context

Mood disorder

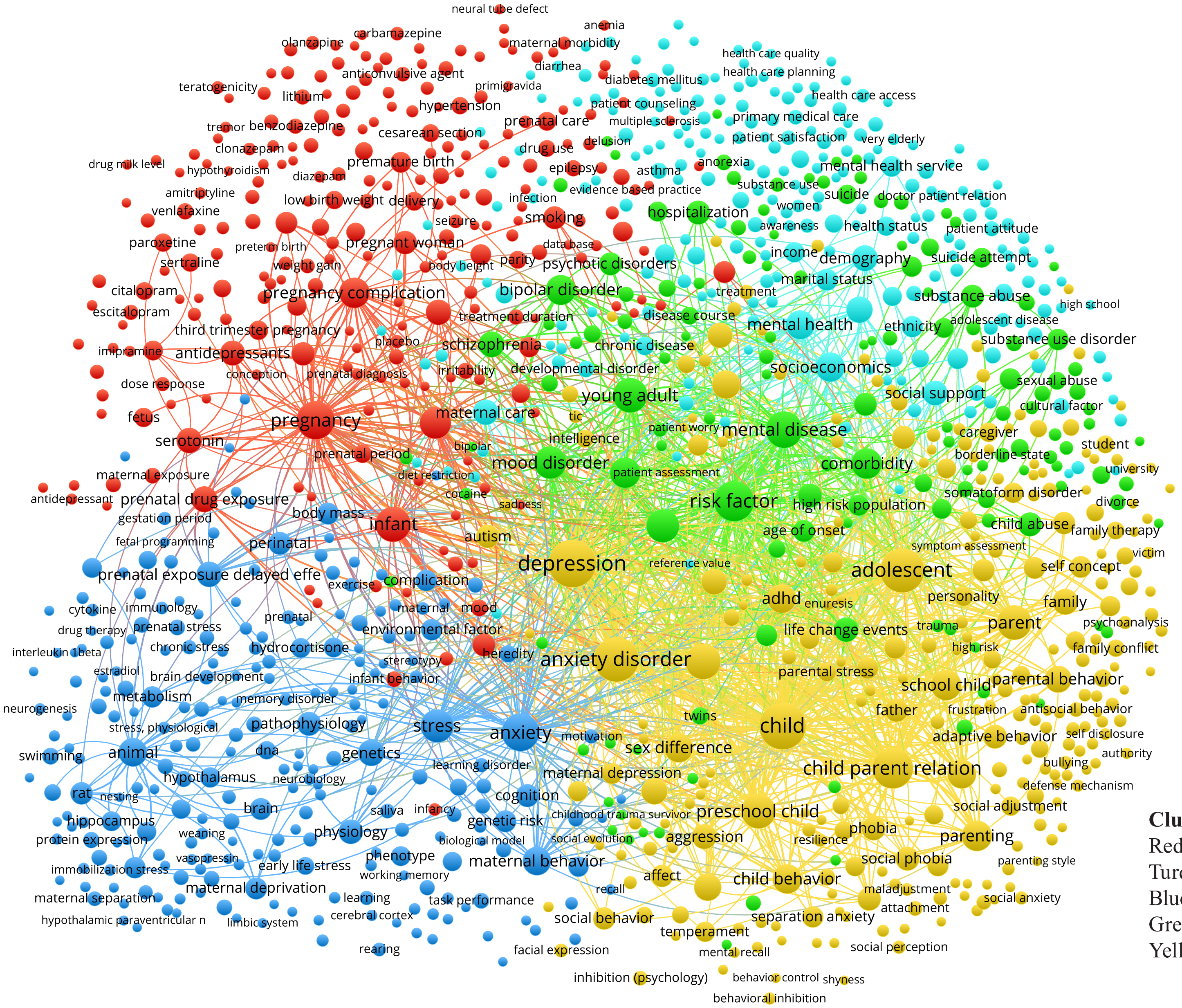

Clusters:

Red: Expectant mothers and early motherhood

Turquoise: SES and support practices

Blue: Biomedical research

Green: Diagnoses, symptoms and treatment

Yellow: Child – parent interaction and context

Personality disorder

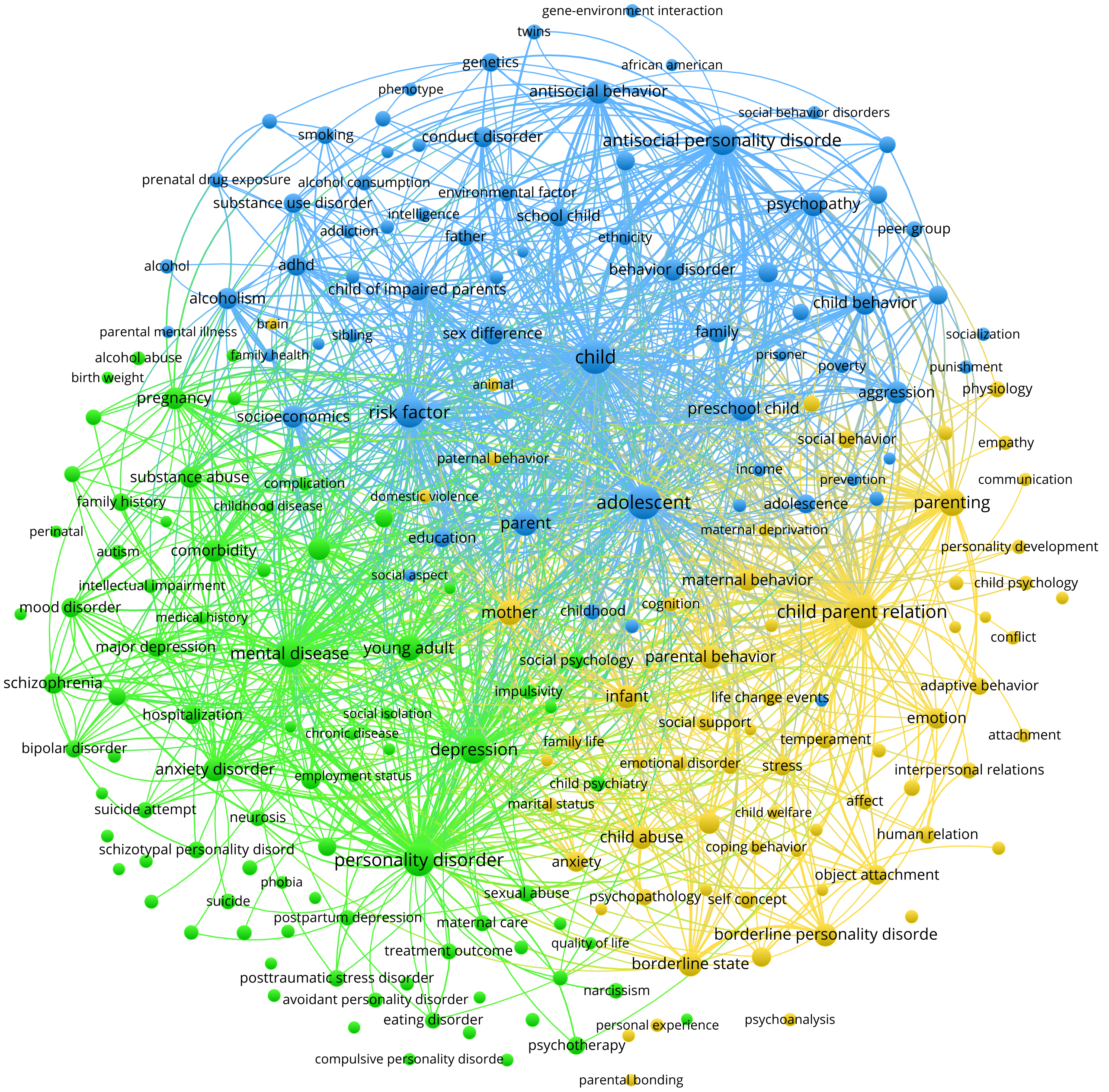

Clusters:
Blue: Biomedical research
Green: Diagnoses, symptoms and treatment
Yellow: Child – parent interaction and context

Psychotic disorder

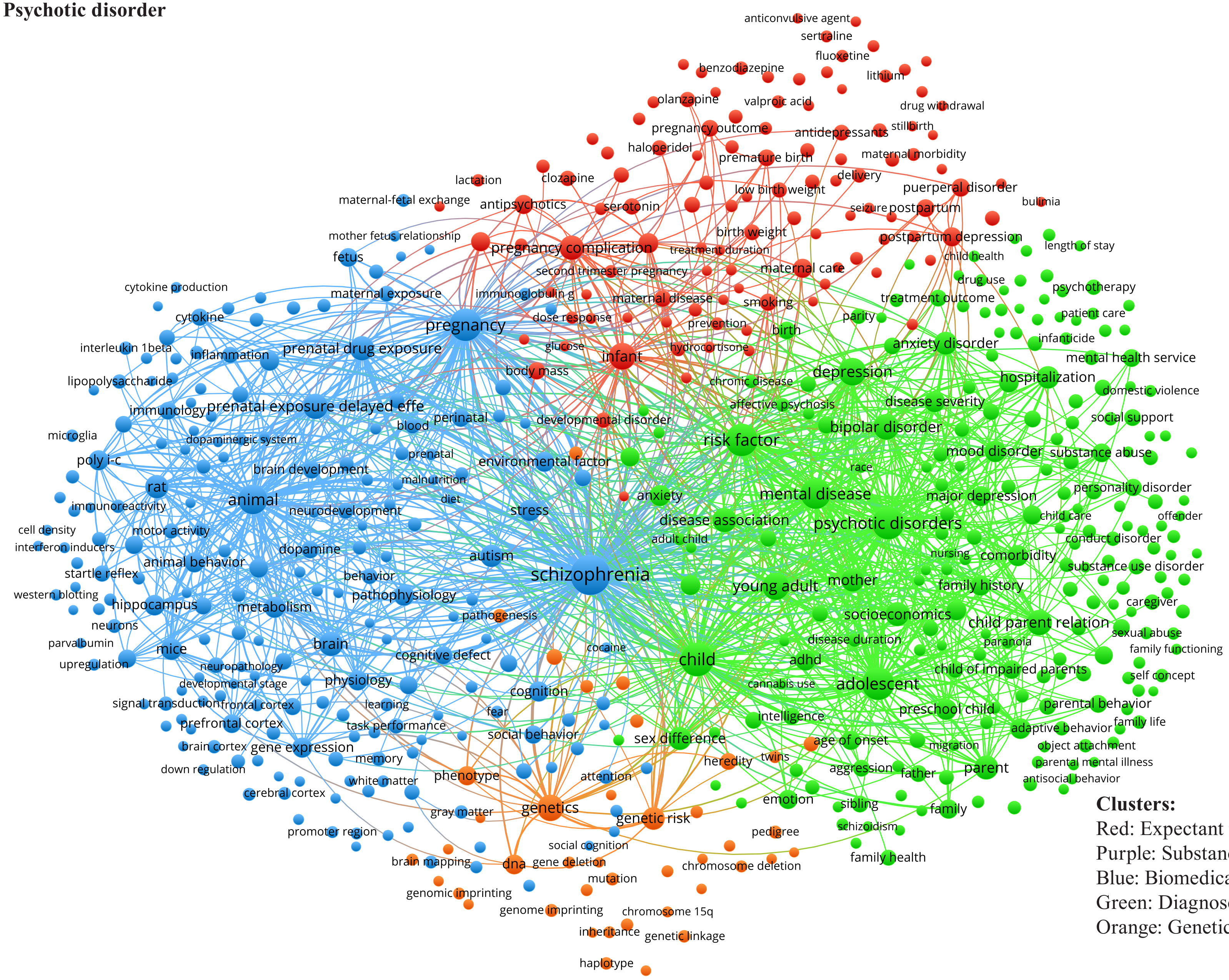

Clusters:
Red: Expectant mothers and early motherhood
Purple: Substance use and abuse
Blue: Biomedical research
Green: Diagnoses, symptoms and treatment
Orange: Genetics

Substance use disorder

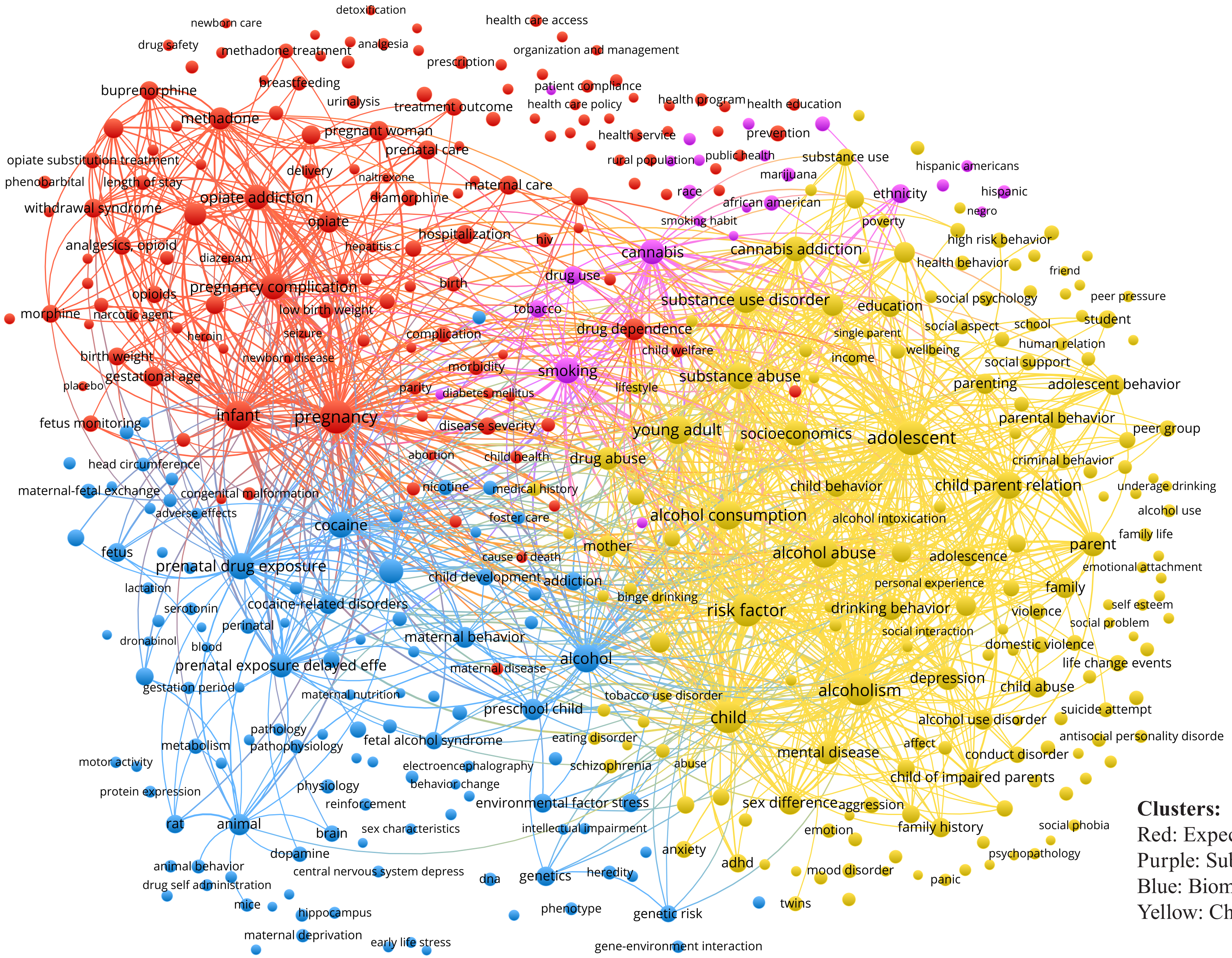

Clusters:

Red: Expectant mothers and early motherhood

Purple: Substance use and abuse

Blue: Biomedical research

Yellow: Child – parent interaction and context

Generic psychiatric terms

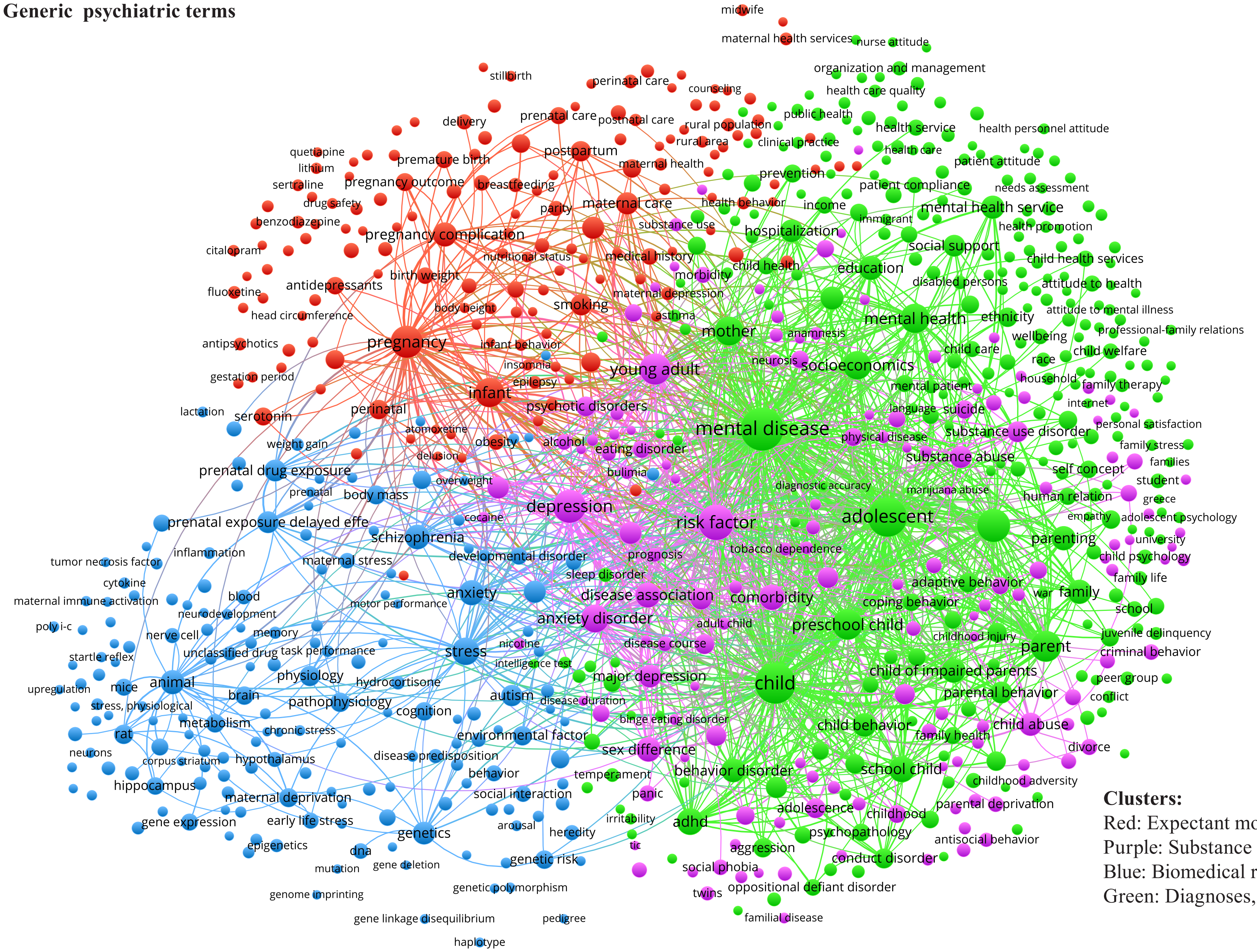

Clusters:
Red: Expectant mothers and early motherhood
Purple: Substance use and abuse
Blue: Biomedical research
Green: Diagnoses, symptoms and treatment

Complete table 2: Number of keyword occurrences in each of the research areas

Keywords that occur in more than 30 articles (n=1332), in the combined search, are listed in the column to the left, and the number of instances in the second column. In the remaining columns, we have listed the number of occurrences for each of the research areas. Cells that are empty, indicate there are fewer than 15 articles with the given keyword in that area.

	Research area/ disorder group							
	**Combined Search**	**Substance Use**	**Behavioral syndromes**	**Personality**	**Psychotic**	**Mood**	**Anxiety**	**Generic Psychiatric**
**Keyword**								
abnormalities, drug-induced	52	18	..	..	..	17	..	..
abnormalities, multiple	31	..	..	..	..	..	..	..
abortion	61	17	21	..	..	..	18	..
abortion, spontaneous	32	..	..	..	..	..	..	..
absenteeism	37	..	16	..	..	..	19	..
abuse	86	17	..	..	..	22	..	32
academic achievement	304	55	64	23	43	60	66	80
acculturation	42	..	..	..	..	..	..	..
achievement	61	..	15	..	..	18	16	..
acoustic stimulation	46	..	..	..	26	..	..	..
acquired immune deficiency syndrome	43	..	..	..	..	..	..	16
actimetry	94	..	91	..	..	..	..	..
activities of daily living	45	..	17	..	..	..	..	..
adaptation	68	..	21	..	..	15	..	15
adaptive behavior	662	44	129	52	48	181	183	182
addiction	234	110	18	30	26	59	35	71
adenoidectomy	34	..	33	..	..	..	..	..
adenotonsillectomy	31	..	31	..	..	..	..	..
adhd	1204	110	211	105	179	359	410	515
adjustment	68	..	..	..	..	15	20	19
adjustment disorder	60	..	..	19	17	27	38	23
adolescence	652	147	112	73	80	162	129	192
adolescent	6093	941	1165	543	771	1616	1485	1947
adolescent behavior	564	165	98	72	24	124	98	140
adolescent development	55	16	..	..	..	..	..	..
adolescent disease	99	..	31	..	..	37	42	26
adolescent health	60	..	20	..	..	..	15	18
adolescent pregnancy	70	..	23	..	..	17	..	21
adolescent psychology	106	..	17	..	..	28	23	28
adoption	70	..	..	..	..	15	..	24
adrenocorticotropic hormone	43	..	..	..	..	15	..	..
adult child	97	15	..	..	26	28	25	39
adult survivors of child abuse	91	18	..	..	..	30	28	46
adult survivors of child adverse events	41	..	..	..	..	18	..	18
adverse effects	115	24	28	..	15	17	..	33
adverse outcome	98	22	24	..	..	28	20	31
affect	461	46	87	45	27	171	126	94
affective neurosis	69	..	..	..	22	49	20	20
affective psychosis	46	..	..	..	46	26	..	..

african american	224	62	43	16	..	58	33	57
african continental ancestry group	40	..	..	..	..	..	..	..
age of onset	461	70	72	27	133	185	115	154
aggression	704	76	116	130	84	200	229	165
aggressiveness	40	..	..	..	..	..	..	..
agitation	33	..	..	..	..	..	..	..
agoraphobia	96	15	17	..	..	46	90	38
alcohol	580	456	29	31	35	55	54	84
alcohol abstinence	31	28	..	..	..	..	..	..
alcohol abuse	560	561	28	25	37	55	47	88
alcohol consumption	801	523	83	39	40	110	94	131
alcohol dependence	40	40	..	..	..	..	..	..
alcohol intoxication	58	47	..	..	..	..	..	..
alcohol use	41	36	..	..	..	..	..	..
alcohol use disorder	149	136	..	..	..	22	21	31
alcoholism	859	652	63	104	76	164	135	224
alexithymia	35	..	..	..	..	..	17	..
algorithm	33	..	..	..	..	..	..	..
allergy	30	..	16	..	..	..	..	..
alzheimer disease	38	..	..	..	19	16	..	..
ambulatory care	75	..	16	..	..	26	..	25
amfebutamone	73	..	17	..	15	41	28	20
amitriptyline	40	..	..	..	..	26	..	..
amphetamine	131	65	..	..	48	..	..	22
amygdala	134	..	..	..	28	53	38	32
analgesia	72	27	18	..	..	..	19	..
analgesics, opioid	116	114	..	..	..	..	..	..
anamnesis	147	24	39	..	32	44	40	42
anemia	80	..	40	..	..	15	..	..
angelman syndrome	34	..	22	..	..	..	..	..
anhedonia	54	..	15	..	..	30	..	15
animal	1611	193	194	22	556	310	296	387
animal behavior	502	44	38	..	153	104	132	123
anorexia	546	..	546	23	24	39	61	65
antenatal depression	100	..	40	..	..	46	30	18
anterior cingulate	32	..	..	..	..	..	..	..
anthropometry	56	..	32	..	..	..	..	..
antibiotic agent	49	..	16	..	..	..	..	..
anticonvulsants	117	..	..	..	..	49	33	..
antidepressants	487	21	105	16	66	295	130	127
antipsychotics	168	..	..	..	126	50	26	45
antisocial behavior	248	44	..	163	20	42	35	64
antisocial personality disorder	362	35	..	362	..	51	33	59
anxiety	2096	86	397	89	173	534	1285	435
anxiety disorder	2577	107	279	147	231	928	2565	627
anxiolytic agent	67	..	..	..	..	19	34	24
apgar score	186	48	48	..	33	44	40	39
apoptosis	31	..	..	..	..	..	..	..
aripiprazole	38	..	..	..	25	17	..	..
arousal	152	..	72	..	..	44	40	27

asian	57	..	20	..	..	18	..	..
asian continental ancestry group	82	..	25	..	16	26	..	21
asperger syndrome	60	..	..	..	16	..	32	20
association	66	..	..	..	..	..	..	..
asthma	186	..	75	..	16	42	67	39
atopic dermatitis	39	..	19	..	..	..	18	..
attachment	166	..	44	27	20	33	43	36
attention	240	20	48	16	48	51	56	58
attention disturbance	78	..	18	..	..	16	30	19
attitude of health personnel	67	..	18	..	..	..	..	26
attitude to health	370	45	124	..	29	65	68	94
attitude to mental illness	34	..	..	..	..	..	..	30
auditory stimulation	30	..	..	..	..	..	..	..
authority	50	..	..	..	..	..	..	..
autism	682	..	116	34	226	159	196	249
autism spectrum disorders	38	..	..	..	..	..	15	..
automutilation	147	20	44	28	29	45	48	47
autonomic nervous system	34	..	..	..	..	..	..	..
autopsy	34	16	..	..	..	..	..	..
avoidance behavior	171	..	20	17	27	40	81	27
avoidance learning	41	..	..	..	..	..	15	..
avoidant personality disorder	34	..	..	34	..	..	19	..
awareness	93	15	30	..	..	15	17	22
backache	31	..	24	..	..	..	..	..
behavior	410	41	92	18	66	79	93	99
behavior change	153	17	34	..	35	21	40	32
behavior control	38	..	..	..	..	..	15	..
behavior disorder	1010	94	174	115	130	286	275	353
behavior problems	37	..	..	..	..	..	..	..
behavior therapy	203	16	72	15	..	42	74	29
behavior, addictive	36	18	..	..	..	..	..	..
behavioral inhibition	34	..	..	..	..	..	31	..
behavioral symptoms	30	..	..	..	..	..	..	..
behaviour	43	..	..	..	..	..	..	..
benzodiazepine	160	49	31	..	39	49	50	43
bereavement	102	..	..	..	18	41	25	40
binge drinking	54	44	..	..	..	..	..	..
binge eating	32	..	31	..	..	..	..	..
binge eating disorder	133	..	133	..	..	..	18	25
biological marker	129	..	27	..	21	42	29	17
biological model	45	..	..	..	..	..	..	..
biomarkers	42	..	..	..	..	17	..	..
bipolar disorder	704	40	66	54	351	683	168	272
birth	523	64	143	..	102	115	97	148
birth defect	37	..	..	..	..	..	..	..
birth weight	468	103	113	18	70	99	78	107
blood	291	17	75	..	61	84	61	69
blood level	33	..	..	..	..	..	..	..
blood pressure	68	..	30	..	..	18	16	..
body build	30	..	26	..	..	..	..	..

body height	149	33	68	..	20	17	..	23
body image	180	..	163	..	..	..	26	..
body mass	957	77	567	..	94	130	135	171
borderline personality disorder	144	..	..	144	..	24	15	33
borderline state	193	..	18	165	17	49	43	53
brain	684	74	54	17	290	163	110	158
brain chemistry	31	..	..	..	..	..	..	..
brain cortex	78	..	..	..	40	15	..	..
brain derived neurotrophic factor	151	..	..	..	35	55	37	47
brain development	207	19	..	..	99	38	32	56
brain disease	39	..	..	..	..	..	..	..
brain hemorrhage	35	..	..	..	..	..	..	..
brain injury	32	..	..	..	..	..	..	..
brain level	35	..	..	..	..	..	..	..
brain mapping	77	..	..	..	36	19	..	..
breastfeeding	447	53	239	..	43	98	67	64
bulimia	301	17	301	19	15	31	45	49
bullying	85	..	..	..	..	20	34	21
buprenorphine	128	129	..	..	..	..	..	..
c reactive protein	45	..	..	..	20	16	..	..
calcium	33	..	..	..	..	..	..	..
caloric intake	34	..	25	..	..	..	..	..
cancer	47	..	..	..	..	..	..	..
cannabinoid	52	27	..	..	20	..	..	..
cannabis	360	312	15	..	38	26	18	44
cannabis addiction	302	305	..	..	28	22	17	43
cannabis use	139	112	..	..	23	..	..	18
carbamazepine	83	..	27	..	28	42	20	16
cardiovascular disease	67	..	23	..	..	21	..	16
cardiovascular risk	35	..	21	..	..	..	..	..
caregiver	437	40	109	27	61	89	100	151
caregiver burden	54	..	..	..	..	..	18	..
catchment area (health)	35	..	..	..	..	..	..	..
caucasian	224	58	..	..	..	37	48	..
caudate nucleus	33	..	..	..	17	..	..	..
cause of death	105	23	..	..	27	26	15	50
cell count	37	..	..	..	18	..	..	..
cell density	30	..	..	..	19	..	..	..
cell proliferation	47	..	..	..	16	..	..	..
central nervous system	32	..	..	..	..	..	..	..
central nervous system depressants	34	25	..	..	..	..	..	..
central stimulant agent	134	47	26	..	19	31	24	39
cerebellum	48	..	..	..	24	..	..	..
cerebral cortex	69	..	..	..	33	19	..	..
cerebral palsy	66	..	31	..	..	..	..	..
cerebrovascular accident	30	..	..	..	..	15	..	..
cesarean section	331	44	127	..	50	66	71	53
chemically induced disorder	45	..	..	..	..	..	..	..
child	6896	710	1335	533	866	1792	1862	2179
child abuse	825	156	91	141	79	245	173	325

child anxiety	59	..	..	..	..	..	60	..
child behavior	1468	164	330	141	81	358	408	364
child behavior disorders	575	41	91	67	31	209	148	181
child care	350	42	110	24	28	69	64	95
child death	30	..	..	..	..	..	..	..
child development	698	86	175	50	57	179	157	188
child development disorders, pervasive	112	..	..	..	21	34	33	50
child growth	78	21	26	..	..	..	..	..
child health	320	35	88	..	22	68	79	114
child health services	203	21	43	..	..	39	34	93
child hospitalization	43	..	..	..	..	..	..	..
child nutrition	96	..	77	..	..	..	..	..
child of impaired parents	1044	162	86	133	117	519	262	382
child parent relation	4255	432	958	512	344	1172	1091	1128
child protection	43	..	..	..	..	..	..	19
child psychiatry	422	19	57	38	64	127	161	186
child psychology	225	..	35	31	..	63	66	74
child sexual abuse	237	43	30	36	28	84	68	81
child welfare	193	39	23	19	16	37	31	84
childhood	404	51	75	44	66	116	107	142
childhood adversity	97	..	..	..	..	45	24	49
childhood cancer	43	..	..	..	..	..	20	..
childhood disease	351	17	89	20	47	106	141	121
childhood injury	63	..	..	..	..	25	19	21
childhood obesity	72	..	50	..	..	..	..	..
childhood trauma	31	..	..	..	..	..	..	..
childhood trauma survivor	46	..	..	..	..	18	..	19
children of alcoholics	37	27	..	..	..	..	..	..
chlorpromazine	49	..	..	..	32	15	..	..
choice behavior	34	..	..	..	..	..	..	..
chromosome 15	64	..	..	..	..	..	..	..
chromosome aberration	32	..	..	..	..	..	..	..
chromosome deletion	77	..	22	..	27	..	15	24
chromosome duplication	32	..	..	..	..	..	..	..
chronic disease	270	..	57	21	34	101	69	91
chronic pain	45	..	..	..	..	..	16	..
chronic stress	78	..	..	..	..	38	23	27
cingulate gyrus	48	..	..	..	22	..	..	..
circadian rhythm	112	..	64	..	..	30	18	..
citalopram	140	..	29	..	25	95	46	28
clinical effectiveness	110	..	30	..	..	23	51	21
clinical examination	42	..	17	..	..	..	..	..
clinical global impression scale	41	..	..	..	..	..	20	..
clinical outcome	38	..	..	..	..	..	..	..
clinical practice	118	..	37	..	..	23	25	36
clinical protocol	67	..	27	..	..	18	22	..
clinical research	52	..	15	..	..	..	..	16
clomipramine	47	..	..	..	..	22	23	..
clonazepam	58	..	22	..	..	27	22	..
clonidine	41	..	..	..	..	..	..	..

clozapine	74	..	..	..	64	17	..	22
cocaine	398	360	18	..	16	22	15	30
cocaine dependence	281	282	..	..	..	..	..	..
cocaine-related disorders	144	138	..	..	..	..	..	..
cognition	659	57	117	35	153	160	156	161
cognition disorders	174	..	23	..	67	53	26	37
cognitive defect	281	21	52	17	110	59	40	67
cognitive development	90	15	21	..	..	21	17	25
cognitive therapy	383	19	74	27	29	129	222	57
combined modality therapy	66	..	..	..	..	19	19	16
communication	299	32	69	23	34	59	76	74
communication disorder	33	..	..	..	..	..	..	..
community	85	..	20	..	..	16	17	36
community care	69	..	17	..	..	16	..	32
community mental health services	60	..	..	..	..	16	..	34
comorbidity	1155	148	224	128	174	499	479	449
comparative genomic hybridization	37	..	..	..	..	..	..	..
competence	64	..	..	..	..	..	18	19
complication	571	67	196	27	80	138	142	153
comprehension	42	..	..	..	..	..	..	15
compulsion	60	..	..	..	..	..	35	..
conception	49	..	..	..	16	17	..	..
conditioning, classical	87	30	..	..	18	..	20	..
conduct disorder	462	75	43	98	51	165	170	200
conflict	157	..	37	26	..	50	34	39
confusion	37	..	..	..	..	..	..	..
congenital heart malformation	49	..	16	..	..	19	..	..
congenital malformation	126	32	29	..	38	39	19	31
constipation	79	..	50	..	..	..	19	..
consultation	106	..	31	..	..	18	29	35
contraception	31	..	..	..	..	..	..	..
convalescence	35	..	..	..	..	..	..	..
cooperation	55	..	16	..	..	..	..	17
cooperative behavior	41	..	..	..	..	..	..	..
coping behavior	450	44	86	34	48	115	140	134
copy number variation	43	..	..	..	25	..	..	..
corpus striatum	82	..	..	..	41	..	..	17
correlation coefficient	37	..	..	..	..	..	..	..
corticosteroid	34	..	..	..	..	..	..	..
corticosterone	207	..	33	..	45	58	53	63
corticotropin	149	..	33	..	..	54	40	28
cortisol	101	..	17	..	..	43	36	23
cost effectiveness analysis	42	..	..	..	..	..	..	15
cost of illness	99	..	20	..	17	29	19	37
coughing	42	..	32	..	..	..	..	..
counseling	101	18	34	..	..	21	17	16
cpg island	33	..	..	..	..	15	..	..
crime victims	63	..	..	..	..	21	20	19
criminal behavior	238	59	15	78	28	45	35	90
criminals	31	..	..	..	..	..	..	16

cross-cultural comparison	104	..	23	..	..	34	24	37
crying	95	..	62	..	..	..	19	..
cultural anthropology	88	..	23	..	..	15	16	22
cultural factor	211	21	57	..	25	58	43	51
culture	125	..	36	..	..	16	30	27
custody	36	..	..	..	..	..	..	17
cytokine	142	..	..	..	88	24	..	30
cytokine production	31	..	..	..	21	..	..	..
daily life activity	73	..	28	..	..	16	19	18
day care	35	..	15	..	..	..	..	..
daytime somnolence	94	..	91	..	..	..	..	..
death	102	..	19	..	16	34	30	47
defense mechanism	73	..	..	23	..	19	28	..
delivery	407	65	162	..	47	104	72	78
delusion	60	..	..	..	53	16	..	15
dementia	32	..	..	..	..	..	..	..
demography	1050	204	202	69	131	296	218	328
dentate gyrus	63	..	..	..	27	18	..	19
dependent personality disorder	80	..	..	80	..	..	15	..
depression	4431	254	824	290	442	2364	1616	1095
depression assessment	36	..	..	..	..	17	..	..
developing country	73	..	..	..	..	25	16	19
developmental delay	35	..	15	..	..	..	..	..
developmental disorder	346	42	91	..	64	66	51	115
developmental stage	59	..	..	..	16	..	..	16
dexamethasone	45	..	..	..	..	..	..	..
dexamphetamine	31	..	..	..	..	..	..	..
diabetes mellitus	164	18	55	..	34	41	32	36
diabetes, gestational	47	..	27	..	..	..	..	..
diagnosis, dual (psychiatry)	39	21	..	..	..	..	..	26
diagnostic accuracy	48	..	..	..	..	..	..	17
diagnostic procedure	37	..	..	..	..	..	..	..
diagnostic value	31	..	..	..	..	..	..	..
diamorphine	90	76	..	..	..	..	..	..
diarrhea	89	..	59	..	..	17	16	..
diazepam	62	17	16	..	..	..	21	..
diet	143	..	90	..	15	..	15	20
diet restriction	97	..	75	..	..	..	..	..
diet therapy	41	..	27	..	..	..	..	..
dietary intake	67	..	34	..	..	..	..	..
dietary supplements	88	..	15	..	..	18	..	..
disabled children	47	..	..	..	..	..	..	15
disabled persons	110	..	15	..	15	32	20	56
disaster	34	..	..	..	..	..	..	17
disease association	1561	154	435	124	300	453	443	438
disease course	252	23	49	25	77	92	59	71
disease duration	139	15	37	..	43	42	33	20
disease predisposition	226	21	38	..	67	69	56	61
disease progression	56	..	..	..	..	21	15	16
disease severity	1069	94	284	65	179	352	347	272

disease susceptibility	50	..	..	..	..	20	15	15
disease transmission	53	..	..	..	16	18	..	..
disruptive behavior	115	..	17	..	20	66	59	45
dissociative disorder	42	..	..	..	..	..	88	..
distress syndrome	353	27	77	16	26	73	163	80
divorce	219	49	22	28	19	49	44	96
dizygotic twins	45	..	..	..	..	..	..	..
dizziness	45	..	32	..	..	..	..	..
dna	333	39	35	..	138	97	53	92
docosahexaenoic acid	38	..	19	..	..	..	..	..
doctor patient relation	98	..	23	..	..	20	29	29
domestic violence	269	73	32	23	26	68	55	111
dopamine	235	59	21	..	95	41	29	45
dopaminergic system	45	..	..	..	24	..	..	..
dose response	196	82	32	..	32	43	24	33
down regulation	51	..	..	..	21	..	..	18
down syndrome	61	..	23	..	..	..	..	..
drinking behavior	338	251	22	16	..	28	30	48
drowsiness	33	..	22	..	..	..	..	..
drug abuse	320	241	21	19	27	36	35	67
drug administration schedule	41	..	..	..	..	..	..	..
drug blood level	90	19	16	..	19	45	15	..
drug dependence	333	198	28	40	58	88	77	113
drug dependence treatment	67	50	..	..	..	..	..	15
drug dose increase	53	17	16	..	..	..	..	..
drug dose reduction	39	..	..	..	..	..	..	..
drug dose titration	32	..	..	..	..	..	..	..
drug effect	504	105	58	..	138	114	81	115
drug efficacy	158	21	60	..	32	38	32	30
drug exposure	92	34	..	..	17	36	..	22
drug mechanism	35	..	..	..	..	..	..	..
drug megadose	46	15	15	..	..	..	..	..
drug misuse	37	25	..	..	..	..	..	..
drug monitoring	31	..	..	..	..	..	..	..
drug response	39	..	..	..	..	..	..	..
drug safety	158	24	61	..	25	45	31	26
drug self administration	31	25	..	..	..	..	..	..
drug tolerability	39	..	27	..	..	..	..	..
drug use	294	125	43	..	28	58	53	70
drug withdrawal	90	18	34	..	16	24	24	15
dyspnea	43	..	28	..	..	..	..	..
dysthymic disorder	129	..	16	24	21	97	67	40
early diagnosis	81	..	25	..	..	21	..	22
early intervention	78	..	..	..	19	16	20	28
early life stress	227	20	..	..	25	69	66	96
eating	253	18	178	..	..	28	33	26
eating disorder	915	38	902	46	52	104	136	124
eating habit	74	..	65	..	..	..	..	..
echography	39	..	19	..	..	..	..	..
eclampsia	37	..	..	..	..	..	..	..

economic aspect	43	..	16	..	..	..	..	15
economics	69	..	17	..	..	..	16	29
eczema	30	..	15	..	..	..	..	..
education	1261	191	318	77	147	269	256	380
education program	32	..	..	..	..	..	..	..
ego development	50	..	..	21	..	..	..	..
electrocardiography	30	..	..	..	..	..	..	..
electroconvulsive therapy	47	..	..	..	23	33	..	18
electroencephalography	163	21	57	..	37	52	31	28
embryo	53	..	..	..	24	..	..	..
emergency service, hospital	37	..	..	..	..	..	..	15
emergency ward	56	..	..	..	..	..	15	17
emigrants and immigrants	67	..	23	..	..	..	..	20
emigration and immigration	32	..	..	..	..	..	..	..
emotion	1018	51	223	102	125	302	303	250
emotional abuse	93	15	..	..	..	28	22	41
emotional attachment	229	21	53	44	22	55	64	45
emotional disorder	365	16	68	40	35	96	119	146
emotional stability	74	..	23	..	..	19	20	19
empathy	82	..	..	30	..	21	..	15
employment status	332	56	68	20	57	85	68	125
enuresis	86	..	51	..	..	17	27	19
environment	165	23	18	..	39	40	32	39
environmental factor	621	126	90	51	149	125	128	188
environmental stress	36	..	..	..	..	..	..	..
enzyme activity	51	..	..	..	16	..	..	..
enzyme linked immunosorbent assay	63	..	..	..	23	..	..	15
epigenetics	159	17	..	..	60	48	33	48
epilepsy	176	..	57	..	31	60	41	47
escitalopram	78	..	18	..	..	57	29	17
estradiol	32	..	..	..	..	..	..	..
estrogen	33	..	..	..	..	..	..	..
ethnic difference	109	25	28	..	..	20	24	27
ethnicity	662	126	170	35	65	156	104	178
european continental ancestry group	132	30	35	..	..	20	17	34
evaluation	88	..	25	..	..	16	20	25
evaluation study	46	..	..	..	..	..	..	19
evidence based medicine	85	..	21	..	..	18	19	15
evidence based practice	47	..	..	..	..	..	16	..
executive function	91	..	..	..	29	25	22	21
exercise	125	..	65	..	..	22	30	19
exon	33	..	..	..	..	..	..	..
expectation	47	..	18	..	..	..	21	..
exploratory behavior	146	..	..	..	49	34	37	41
exposure to violence	32	..	..	..	..	..	..	..
extraversion	44	..	..	..	..	..	20	..
facial expression	83	..	17	..	..	29	27	17
familial disease	70	..	..	..	19	24	22	24
family	1125	172	187	97	134	281	231	373

family attitude	48	..	19	..	..	..	..	..
family characteristics	191	29	26	15	18	49	30	79
family conflict	211	19	32	32	21	84	47	64
family counseling	53	..	..	..	..	..	..	16
family environment	33	..	..	..	..	..	..	15
family functioning	221	18	45	17	19	70	72	69
family health	327	37	53	24	65	93	64	115
family history	606	134	104	61	150	211	140	221
family income	35	..	..	..	..	..	..	..
family interaction	52	..	..	..	..	..	..	..
family life	259	39	60	30	40	54	75	64
family planning	34	..	..	..	..	..	..	..
family stress	96	..	20	..	..	24	31	31
family therapy	260	..	82	16	29	67	70	58
father	638	93	130	67	81	185	169	187
fatigue	180	..	125	..	..	34	42	..
fear	293	..	47	17	30	52	172	61
feces incontinence	31	..	..	..	..	..	18	..
feeding behavior	427	..	367	..	..	31	50	33
feeding difficulty	34	..	26	..	..	..	..	..
female infertility	33	..	15	..	..	..	..	..
fertilization in vitro	33	..	..	..	..	..	..	..
fetal alcohol spectrum disorders	46	33	..	..	..	..	..	..
fetal alcohol syndrome	124	91	..	..	..	..	..	23
fetal blood	50	..	..	..	..	20	..	..
fetus	495	126	77	..	114	109	75	75
fetus death	41	..	..	..	..	..	..	..
fetus echography	43	..	..	..	..	..	..	..
fetus monitoring	327	79	86	..	61	80	55	44
fever	116	..	59	..	16	..	30	..
financial management	31	..	..	..	..	..	..	..
first trimester pregnancy	261	44	57	..	39	74	65	52
fluorescence in situ hybridization	34	..	16	..	..	..	..	..
fluoxetine	216	..	52	..	29	128	74	43
fluvoxamine	71	..	17	..	..	38	27	..
folic acid	93	..	32	..	31	17	..	..
food	30	..	16	..	..	..	..	..
food habits	47	..	30	..	..	..	..	..
food preference	86	..	47	..	..	..	18	15
forecasting	37	..	..	..	..	..	..	..
forensic psychiatry	40	..	..	..	..	..	..	22
foster care	92	32	..	..	..	..	..	36
friend	64	15	..	..	..	..	15	..
frontal cortex	57	..	..	..	30	..	..	..
frontal lobe	62	..	..	..	25	26	..	..
frustration	39	..	..	..	..	..	16	..
functional assessment	47	..	..	..	..	16	..	..
functional disease	83	..	17	..	..	31	40	26
functional laterality	51	..	..	..	31	..	..	..
functional status	30	..	..	..	..	..	..	..

gamma interferon	40	..	..	..	22	..	..	..
gastroesophageal reflux	34	..	26	..	..	..	..	..
gastrointestinal disease	39	..	15	..	..	..	..	..
gastrointestinal symptom	49	..	30	..	..	..	..	..
gender differences	44	..	..	..	..	..	..	..
gender identity	68	..	16	..	..	19	15	18
gene deletion	67	..	22	..	27	..	..	15
gene expression	420	32	39	..	158	103	71	119
gene identification	30	..	..	..	..	..	..	..
gene interaction	32	..	..	..	..	..	..	..
gene linkage disequilibrium	49	..	..	..	29	16	..	18
gene-environment interaction	184	30	..	23	43	46	35	58
general practice	33	..	..	..	..	..	..	..
genetic disorder	44	..	..	..	..	..	..	..
genetic epigenesis	93	15	..	..	30	26	20	32
genetic linkage	52	..	..	..	31	19	..	..
genetic polymorphism	89	..	..	..	32	33	16	21
genetic risk	565	88	41	38	225	191	104	148
genetic screening	52	..	..	..	23	..	..	..
genetic variation	35	..	..	..	16	..	..	..
genetics	1148	138	155	68	381	307	224	313
genome imprinting	67	..	..	..	33	15	..	16
genomic dna	41	..	..	..	16	..	..	..
genomic imprinting	51	..	..	..	27	..	..	..
geographic distribution	34	..	..	..	..	..	..	..
gestation period	130	28	15	..	41	27	22	25
gestational age	787	145	192	15	126	176	160	164
gilles de la tourette syndrome	39	..	..	..	..	..	34	..
glucocorticoid	135	..	18	..	22	49	44	38
glucocorticoids	43	..	..	..	..	18	..	..
glucose	83	..	44	..	17	..	17	..
glutamic acid	46	..	..	..	21	..	..	..
government	30	..	..	..	..	..	..	16
grandparent	31	..	..	..	..	..	..	..
gray matter	59	..	..	..	39	..	..	..
grief	53	..	..	..	..	18	..	16
grooming	47	..	..	..	..	..	16	..
group psychology	34	..	..	..	..	..	..	..
group therapy	78	..	15	..	..	29	36	18
growth	37	..	17	..	..	..	..	..
growth retardation	39	..	..	..	..	..	..	..
growth, development and aging	90	..	..	..	31	17	20	25
guilt	62	..	17	..	..	..	21	..
habituation	40	..	..	..	..	..	..	..
hair	35	20	..	..	..	..	..	..
hallucination	57	..	..	..	47	..	..	15
haloperidol	88	..	15	..	64	26	22	25
handedness	34	..	..	..	25	..	..	..
handicapped child	38	..	..	..	..	..	..	..
haplotype	62	..	..	..	29	20	..	20

happiness	38	..	..	..	..	..	..	..
happy puppet syndrome	41	..	25	..	..	..	..	..
head circumference	98	43	22	..	..	17	..	18
headache	175	..	89	..	15	43	60	32
health behavior	214	53	81	..	..	28	32	45
health belief	41	..	..	..	..	..	17	..
health care	69	..	19	..	..	..	..	23
health care access	149	29	32	..	..	26	30	59
health care cost	71	..	17	..	..	..	..	23
health care delivery	128	20	35	..	..	18	20	48
health care need	127	..	31	..	..	24	15	61
health care personnel	131	21	36	..	..	28	26	43
health care planning	54	..	..	..	..	16	..	16
health care policy	94	23	18	..	..	..	..	34
health care quality	93	..	27	..	..	..	..	42
health care surveys	38	..	17	..	..	..	..	..
health care system	35	..	..	..	..	..	..	..
health care utilization	157	25	34	..	..	35	37	72
health center	43	..	..	..	..	..	..	..
health disparity	36	..	..	..	..	..	..	18
health education	270	28	37	..	15	24	21	28
health hazard	55	16	..	..	..	..	..	..
health insurance	69	15	18	..	..	..	..	27
health personnel attitude	62	..	16	..	..	..	..	26
health practitioner	45	..	..	..	..	..	..	22
health program	175	33	49	..	..	30	33	39
health promotion	99	..	34	..	..	15	..	34
health risk	62	..	..	..	..	..	..	29
health service	223	35	57	..	..	41	28	99
health status	413	39	115	17	21	91	93	144
health status indicators	35	..	..	..	..	..	..	..
hearing impairment	33	..	..	..	..	..	..	..
heart defects, congenital	33	..	..	..	..	..	..	..
heart disease	52	..	16	..	..	15	..	..
heart rate	78	15	17	..	..	20	23	..
help seeking behavior	76	..	16	..	..	22	21	36
hepatitis c	33	22	..	..	..	..	..	..
heredity	245	43	22	23	80	69	59	79
heroin dependence	71	60	..	..	..	..	..	..
heterozygote	55	..	..	..	16	..	..	..
high performance liquid chromatography	37	..	..	..	..	..	..	..
high risk behavior	141	65	..	..	..	18	17	46
high risk patient	145	17	39	..	39	50	31	31
high risk population	440	85	79	26	88	152	81	131
high risk pregnancy	90	17	32	..	16	19	15	17
high school	54	20	..	..	..	..	..	..
hippocampus	426	25	26	..	163	103	83	127
hispanic	161	38	39	..	..	42	33	41
hispanic americans	104	26	25	..	..	26	16	28
histopathology	37	..	15	..	..	..	..	..

history	32	..	..	..	..	..	..	16
hiv	153	56	24	..	..	33	25	47
home	49	..	..	..	..	..	..	..
home care	66	..	17	..	..	..	..	18
home environment	43	..	..	..	..	..	..	..
homelessness	47	15	..	..	..	..	..	22
homicide	70	..	..	..	33	17	..	33
hormone blood level	35	..	..	..	..	..	..	..
hospital	43	..	16	..	..	..	..	..
hospital readmission	34	..	..	..	..	..	..	..
hospitalization	848	122	195	73	220	222	162	305
house calls	39	..	..	..	..	..	..	..
household	99	20	23	..	..	23	20	37
household income	33	..	..	..	..	..	..	..
housing	46	..	..	..	..	..	..	..
hpa axis	55	..	..	..	..	24	20	18
human cell	55	..	..	..	15	..	..	..
human relation	388	35	67	54	41	104	89	120
human tissue	102	30	23	..	19	19	..	..
hunger	35	..	17	..	..	..	..	..
hydrocortisone	263	..	58	..	23	117	90	54
hydrocortisone blood level	65	..	16	..	..	22	25	..
hypertension	339	38	151	..	47	77	62	54
hypertension, pregnancy-induced	46	..	26	..	..	..	..	..
hypoglycemia	52	..	26	..	..	..	..	..
hypomania	31	..	..	..	..	30	..	..
hypophysis adrenal system	74	..	..	..	..	42	21	21
hypothalamus	316	19	59	..	46	121	85	76
hypothyroidism	36	..	15	..	..	..	..	..
illicit drug	126	105	..	..	..	..	..	19
imipramine	36	..	..	..	..	22	..	..
immigrant	67	..	21	..	16	..	..	25
immigration	30	..	..	..	..	..	..	..
immobilization stress	50	..	..	..	..	..	19	16
immune response	106	..	15	..	75	16	..	21
immune system	56	..	..	..	37	..	..	..
immunity	37	..	..	..	25	..	..	..
immunoglobulin g	36	..	..	..	30	..	..	..
immunology	146	..	15	..	102	29	..	34
immunoreactivity	76	..	..	..	40	..	20	..
immunostimulation	41	..	..	..	38	..	..	..
impulsive behavior	53	..	..	..	..	..	..	16
impulsivity	165	28	40	33	19	38	33	54
in situ hybridization	31	..	..	..	..	..	..	..
in vitro study	35	..	..	..	..	..	..	15
income	442	72	114	28	29	132	86	123
individuality	51	..	..	..	..	18	..	..
infancy	88	..	33	..	..	19	24	18
infant	3321	530	1021	151	398	725	587	735
infant behavior	176	..	72	..	..	52	36	32

infant care	59	..	39	..	..	..	..	..
infant disease	80	..	27	..	..	18	16	..
infant feeding	76	..	58	..	..	..	..	..
infant mortality	55	..	..	..	..	..	..	19
infant nutrition	36	..	16	..	..	..	..	..
infant, small for gestational age	50	..	..	..	..	15	..	..
infanticide	50	..	..	..	30	17	..	23
infection	117	..	19	..	64	15	..	23
infertility	30	..	..	..	..	..	..	..
inflammation	151	..	22	..	77	37	17	36
influenza	64	..	..	..	48	..	..	..
information processing	152	26	45	..	20	18	23	45
inheritance	63	..	..	..	27	15	..	15
inhibition (psychology)	97	..	..	..	18	25	43	19
injury	84	..	18	..	..	25	18	27
inpatients	42	..	..	..	..	..	..	30
insomnia	159	..	113	..	16	32	38	27
insulin	52	..	23	..	..	..	..	..
insulin dependent diabetes mellitus	53	..	24	..	..	..	16	..
intellectual impairment	289	24	68	29	72	59	73	116
intelligence	326	41	45	31	92	77	69	101
intelligence test	38	..	..	..	..	..	..	16
intensive care units, neonatal	48	..	18	..	..	..	..	..
intergenerational relations	55	..	..	..	..	16	..	18
intergenerational transmission	38	..	..	..	..	..	..	..
interleukin 10	43	..	..	..	22	..	..	..
interleukin 1beta	62	..	..	..	41	..	..	..
interleukin-6	51	..	..	..	31	..	..	..
internet	104	..	38	..	..	17	34	19
interpersonal relations	271	24	39	45	28	83	61	81
intervention	59	..	16	..	..	..	..	19
intimate partner violence	140	35	32	..	..	33	30	44
intrauterine growth retardation	121	40	24	..	25	24	..	25
intrauterine infection	53	..	..	..	43	..	..	15
iron	55	..	24	..	..	..	..	..
irritability	162	..	77	..	24	56	49	28
ischemic heart disease	36	..	..	..	..	18	..	..
juvenile delinquency	158	46	..	51	..	24	19	47
knowledge	50	..	..	..	..	..	..	19
labor	48	..	16	..	..	..	..	..
labor complication	49	..	..	..	19	..	..	..
labor induction	37	..	..	..	..	..	..	..
lactation	180	25	65	..	31	45	32	27
lamotrigine	63	..	16	..	17	44	17	..
language	83	..	20	..	20	19	..	21
language development	48	..	..	..	..	..	..	16
language disability	67	..	16	..	..	..	..	28
latent period	56	..	..	..	..	..	15	..
learning	135	..	17	..	37	23	37	34
learning disorder	138	..	27	..	36	26	40	48

legal aspect	67	..	..	..	..	..	..	30
leisure	33	..	..	..	..	..	..	..
length of stay	130	58	29	..	20	16	18	36
leptin	35	..	19	..	..	..	..	..
lethargy	31	..	20	..	..	..	..	..
life change events	607	75	86	51	70	251	174	210
life satisfaction	43	..	..	..	..	..	..	..
life stress	113	..	25	..	..	43	33	28
lifespan	76	18	..	..	..	33	28	31
lifestyle	155	35	57	..	..	26	19	30
lifestyle modification	31	..	18	..	..	..	..	..
limbic system	44	..	..	..	16	..	..	..
linkage disequilibrium	36	..	..	..	16	..	..	..
lipid diet	31	..	..	..	..	..	..	..
lipopolysaccharide	78	..	..	..	50	..	..	18
lithium	89	..	..	..	33	68	19	29
locomotion	187	27	15	..	87	28	32	38
loneliness	49	..	15	..	..	..	16	..
long term care	67	..	16	..	15	19	18	16
long term exposure	33	..	..	..	..	..	..	..
lorazepam	46	..	..	..	..	21	19	..
low birth weight	344	71	78	..	60	91	65	92
low drug dose	40	..	..	..	..	..	..	..
maintenance therapy	35	24	..	..	..	..	..	..
major depression	1016	78	118	80	147	887	325	311
maladjustment	89	..	21	..	..	15	27	26
malnutrition	85	..	33	..	16	..	..	19
maltreatment	31	..	..	..	..	..	..	..
manic depressive psychosis	52	..	..	..	52	43	..	..
marijuana	47	45	..	..	..	..	..	..
marijuana abuse	169	171	..	..	..	..	..	17
marijuana smoking	62	57	..	..	..	..	..	..
marital status	484	79	124	35	56	151	118	128
maternal	89	..	28	..	..	22	18	24
maternal anxiety	54	..	..	..	..	..	47	..
maternal attitude	220	18	82	..	..	47	64	44
maternal behavior	1360	168	359	107	154	333	347	294
maternal blood	56	..	..	..	18	17	..	..
maternal care	1007	126	301	44	132	232	230	280
maternal depression	277	..	51	..	..	188	48	49
maternal deprivation	499	38	39	21	104	149	117	164
maternal disease	368	29	80	15	86	125	103	110
maternal exposure	261	78	27	..	68	63	33	54
maternal health	132	15	41	..	..	27	24	47
maternal health service	124	24	..	..	..	23	..	15
maternal immune activation	96	..	..	..	83	..	..	23
maternal mental health	65	..	..	..	..	17	15	33
maternal morbidity	168	31	47	..	36	34	28	49
maternal nutrition	155	18	53	..	44	19	..	32
maternal nutritional physiological phenomena	48	..	16	..	..	..	..	..

maternal obesity	71	..	38	..	16	17	..	17
maternal separation	122	16	..	..	..	36	30	45
maternal stress	314	..	86	..	38	73	116	87
maternal treatment	32	..	..	..	..	..	..	..
maternal-child nursing	37	..	22	..	..	..	..	..
maternal-fetal exchange	162	58	15	..	35	43	18	21
meconium	50	44	..	..	..	..	..	..
medial prefrontal cortex	43	..	..	..	18	..	..	16
medicaid	46	..	..	..	..	..	..	15
medical assessment	63	..	16	..	..	18	..	16
medical decision making	49	..	..	..	..	..	20	17
medical education	39	..	..	..	..	..	..	15
medical history	366	43	106	19	72	110	88	110
medical information	71	..	22	..	..	..	..	28
medical specialist	42	..	15	..	..	..	..	15
melatonin	51	..	37	..	..	..	..	..
memory	147	..	..	..	48	29	38	40
memory disorder	101	..	..	..	46	20	17	24
mental development	57	..	..	..	..	..	..	20
mental disease	3641	266	354	302	623	914	845	2624
mental health	1475	108	263	81	142	370	403	754
mental health service	570	32	73	35	95	147	102	325
mental hospital	83	..	..	..	40	36	..	53
mental illness	98	..	..	..	24	17	..	93
mental instability	32	..	..	..	..	..	..	..
mental patient	155	..	22	18	62	51	36	64
mental performance	37	..	..	..	..	..	..	..
mental recall	39	..	..	..	..	..	16	..
messenger rna	195	..	19	..	69	49	35	58
metabolic disorder	37	..	..	..	17	..	..	..
metabolism	590	62	87	..	179	146	123	157
methadone	222	217	..	..	..	..	..	..
methadone treatment	55	55	..	..	..	..	..	..
methamphetamine	64	47	..	..	..	..	..	..
methylphenidate	94	..	32	..	16	22	25	36
mice	467	37	43	..	196	88	84	115
microcephaly	49	15	17	..	..	..	..	..
microglia	40	..	..	..	30	..	..	..
midwife	90	..	32	..	..	17	..	33
migrant	40	..	16	..	..	..	..	17
migration	73	..	18	..	19	..	..	22
military personnel	31	..	..	..	..	..	..	20
milk, human	48	..	19	..	..	17	..	..
minority group	34	..	..	..	..	..	..	..
mirtazapine	31	..	..	..	..	17	..	..
monitoring	41	..	..	..	..	..	..	..
monotherapy	41	..	16	..	..	15	..	..
mood	156	..	46	..	17	72	47	26
mood change	35	..	15	..	..	19	..	..
mood disorder	919	55	129	80	196	924	345	294

mood stabilizer	48	..	..	..	20	33	..	16
morbidity	310	60	63	..	68	75	47	95
morphine	115	101	..	..	..	..	..	..
mother	2560	208	690	187	260	797	654	682
mother fetus relationship	51	..	..	..	19	..	..	..
mother-infant interaction	30	..	..	..	..	..	..	..
motivation	175	38	28	21	24	33	32	46
motor activity	194	24	37	..	63	30	38	35
motor development	43	..	..	..	..	..	..	..
motor dysfunction	68	..	31	..	20	..	..	..
motor performance	62	..	18	..	17	15	..	17
multimodality cancer therapy	36	..	..	..	..	..	..	..
multipara	46	..	17	..	..	..	..	..
multiple pregnancy	36	..	18	..	..	..	..	..
muscle hypotonia	49	..	25	..	..	..	..	..
mutation	80	..	21	..	35	..	..	16
n methyl dextro aspartic acid receptor	38	..	..	..	23	..	..	..
narcissism	62	..	..	38	..	..	18	..
narcotic agent	32	30	..	..	..	..	..	..
nausea	100	..	68	..	..	..	18	..
needs assessment	54	..	..	..	..	..	..	26
negative syndrome	41	..	..	..	37	..	..	..
neglect	104	18	..	..	..	31	..	39
negro	45	15	..	..	..	..	..	..
neonatal abstinence syndrome	159	141	..	..	..	..	..	..
neonatal intensive care unit	45	..	15	..	..	..	..	..
neonate	33	..	..	..	..	..	..	..
neoplasm	90	..	..	..	..	20	37	..
nerve cell	277	23	32	..	125	50	35	80
nerve cell network	31	..	..	..	..	..	..	..
nerve protein	60	..	..	..	33	19	..	15
nervous system development	127	..	15	..	50	32	22	35
nervousness	30	..	18	..	..	..	..	..
neural pathways	37	..	..	..	..	..	..	..
neural tube defect	34	..	..	..	..	15	..	..
neuroanatomy	36	..	..	..	16	..	..	..
neurobiology	58	..	..	..	16	18	16	15
neurochemistry	35	..	..	..	..	..	..	..
neurodevelopment	104	..	..	..	71	..	..	27
neurodevelopmental disorders	54	..	..	..	23	16	17	15
neuroendocrine system	31	..	..	..	..	..	..	..
neurogenesis	61	..	..	..	25	15	..	19
neuroleptic agent	249	..	30	..	158	94	54	82
neurologic disease	114	..	25	..	40	21	20	30
neurologic examination	58	..	15	..	15	..	..	16
neuromodulation	33	..	..	..	..	..	..	..
neuronal plasticity	40	..	..	..	..	..	..	15
neurons	85	..	..	..	46	..	..	21
neuropathology	43	..	..	..	21	..	..	..
neuropsychiatry	32	..	..	..	17	..	..	..

neuropsychology	46	..	..	..	19	..	..	..
neurosis	120	..	19	43	41	48	55	39
neurotoxicity	38	..	..	..	..	..	..	..
neurotransmission	50	..	..	..	23	..	..	..
newborn care	45	15	..	..	..	..	..	..
newborn disease	92	20	23	..	22	31	18	16
newborn intensive care	79	16	32	..	..	19	15	..
newborn morbidity	33	..	..	..	..	..	..	..
newborn mortality	32	..	..	..	..	..	..	..
nicotine	124	59	16	..	22	26	..	31
night sleep	52	..	49	..	..	..	..	..
nightmare	53	..	39	..	..	..	..	..
non insulin dependent diabetes mellitus	50	..	22	..	16	..	..	..
noradrenalin	52	..	..	..	..	25	..	16
nortriptyline	46	..	15	..	..	28	..	..
nuclear family	70	..	15	..	16	21	..	23
nucleotide sequence	58	..	..	..	26	16	..	..
nucleus accumbens	91	24	..	..	30	19	..	22
nullipara	39	..	19	..	..	..	..	..
nurse	49	..	19	..	..	..	..	20
nurse attitude	56	..	27	..	..	..	..	18
nurse patient relationship	31	..	..	..	..	..	..	15
nurse’s role	65	..	31	..	..	..	..	19
nursing	177	15	66	..	18	27	22	63
nursing assessment	35	..	19	..	..	..	..	..
nutrition	64	..	35	..	..	..	..	..
nutritional status	93	..	48	..	..	..	..	26
obesity	463	32	303	..	37	79	62	85
object attachment	471	25	100	94	41	143	123	116
observation	32	..	..	..	..	..	..	..
observer variation	67	..	18	..	..	17	21	25
obsession	41	..	..	..	..	..	28	..
obsessive compulsive disorder	410	..	74	31	84	101	412	116
obstetric complications	90	..	..	..	50	17	16	21
obstetric labor, premature	30	..	..	..	..	..	..	..
obstetrics	73	..	23	..	18	15	..	16
occupation	81	..	25	..	17	21	15	28
offender	74	18	..	27	17	16	..	38
olanzapine	92	..	..	..	61	38	20	30
omega 3 fatty acid	55	..	22	..	..	..	..	..
open field behavior	31	..	..	..	..	..	..	..
opiate	212	186	..	..	..	..	..	24
opiate addiction	303	307	..	..	..	16	..	23
opiate substitution treatment	71	69	..	..	..	..	..	..
opioid dependence	34	33	..	..	..	..	..	..
opioid use disorder	209	211	..	..	..	..	..	16
opioids	102	97	..	..	..	..	..	..
oppositional defiant disorder	204	..	23	28	30	73	107	107
organ size	41	..	..	..	..	..	..	..
organization and management	146	17	37	..	..	26	16	65

outcome	35	..	..	..	..	..	..	..
outpatient	129	..	19	16	26	50	25	40
outpatient care	150	25	40	..	21	42	34	62
outpatients	35	..	..	..	..	..	..	17
overweight	100	..	75	..	..	..	..	19
oxidative stress	59	..	..	..	26	15	..	..
oxygen	34	..	..	..	..	..	..	..
oxytocin	118	..	36	..	18	19	41	20
oxytocin receptor	34	..	..	..	..	..	..	..
pain	140	19	47	..	..	32	41	20
pain assessment	61	..	..	..	..	..	24	..
panic	236	29	42	16	25	112	214	94
panic disorder	80	..	..	..	..	40	63	26
paracetamol	44	..	..	..	..	..	15	..
paranoia	60	..	..	16	35	..	32	..
parasomnia	58	..	57	..	..	..	..	..
parent	2615	286	577	211	274	622	716	876
parent counseling	141	20	32	..	..	26	38	31
parental attitude	620	79	173	43	52	100	208	150
parental behavior	1075	175	214	158	95	217	336	269
parental bonding	71	..	21	17	..	20	..	..
parental consent	51	..	..	..	..	..	..	..
parental death	51	..	..	..	..	20	..	30
parental depression	40	..	..	..	..	36	..	..
parental deprivation	207	29	..	25	34	88	63	84
parental mental illness	142	..	..	18	16	28	33	107
parental smoking	42	..	..	..	..	..	..	..
parental stress	306	..	95	..	..	64	101	86
parenting	1584	155	281	249	105	407	426	384
parenting stress	63	..	28	..	..	..	16	17
parity	327	44	101	17	63	76	69	82
paroxetine	161	..	38	..	26	97	61	33
passive smoking	42	..	21	..	..	..	..	..
paternal attitude	55	..	17	..	..	16	15	..
paternal behavior	229	45	48	28	32	61	59	50
paternal deprivation	31	..	..	..	..	15	..	..
pathogenesis	109	..	20	..	42	28	24	28
pathology	243	30	38	..	79	51	44	50
pathophysiology	777	46	215	31	168	218	152	187
patient admission	55	..	..	..	19	..	..	29
patient assessment	41	..	..	..	..	..	..	..
patient attitude	188	21	50	..	21	34	53	54
patient care	137	18	40	..	28	26	26	49
patient care team	43	..	15	..	..	..	..	18
patient compliance	305	34	91	..	33	68	71	93
patient counseling	102	22	30	..	..	25	22	15
patient discharge	31	..	..	..	..	..	..	..
patient education	107	..	36	..	..	20	27	27
patient education as topic	51	..	19	..	..	..	..	..
patient monitoring	45	..	19	..	..	..	..	..

patient participation	41	..	..	..	..	..	..	18
patient referral	169	..	56	..	22	42	37	53
patient satisfaction	141	..	46	..	..	32	31	33
patient selection	37	..	..	..	..	..	..	..
pediatric hospital	41	..	17	..	..	..	..	..
pediatric obesity	32	..	24	..	..	..	..	..
pedigree	85	..	..	..	36	25	..	15
peer group	308	80	55	41	16	69	66	57
peer pressure	52	24	..	..	..	..	..	..
perfectionism	49	..	27	..	..	..	23	..
perinatal	589	64	125	16	105	155	156	183
perinatal care	134	18	35	..	18	32	23	58
perinatal depression	97	..	45	..	..	52	27	21
perinatal drug exposure	42	..	..	..	..	..	..	..
peripartum period	31	..	15	..	..	..	..	..
personal autonomy	44	..	..	..	..	..	..	..
personal experience	235	26	42	23	40	72	68	84
personal satisfaction	96	..	39	..	..	22	23	22
personality	339	36	78	111	41	72	103	73
personality development	77	..	..	30	..	23	21	15
personality disorder	434	26	54	434	89	128	112	138
phenobarbital	58	32	16	..	..	..	..	..
phenotype	400	32	63	24	129	95	93	110
phenytoin	32	..	..	..	..	..	..	..
phobia	313	15	26	21	27	70	314	65
physical abuse	177	38	26	..	26	55	47	80
physical activity	138	..	69	..	..	34	27	22
physical disease	68	..	..	..	..	18	22	38
physical examination	81	..	41	..	..	..	16	..
physician	61	..	18	..	..	..	18	16
physician attitude	40	..	..	..	..	..	..	..
physician-patient relations	30	..	..	..	..	..	..	..
physiological stress	51	..	..	..	..	18	17	17
physiology	825	48	210	43	147	220	175	207
pituitary-adrenal system	132	..	17	..	..	68	40	34
placebo	101	15	41	..	..	22	22	17
play	79	..	..	..	..	..	22	..
policy	31	..	..	..	..	..	..	..
poly i-c	155	..	..	..	150	..	..	25
polymerase chain reaction	69	..	..	..	26	20	..	17
polymorphism, genetic	61	..	..	..	..	19	..	20
polysomnography	154	..	151	..	..	..	..	..
population	57	..	..	..	..	15	21	15
positive end expiratory pressure	33	..	29	..	..	..	..	..
positron emission tomography	32	..	..	..	15	..	..	..
postnatal	36	..	..	..	..	..	..	..
postnatal care	156	15	76	..	17	26	18	39
postnatal development	63	..	..	..	18	..	..	15
postoperative period	30	..	16	..	..	..	..	..
postpartum	967	79	507	24	95	255	235	209

postpartum depression	1866	26	1157	40	130	599	369	287
postpartum hemorrhage	43	..	..	..	..	..	..	..
posttraumatic stress disorder	501	44	78	47	57	174	256	199
poverty	294	30	49	30	16	97	47	108
practice guidelines as topic	44	..	..	..	..	..	..	..
prader willi syndrome	71	..	32	..	28	..	..	..
preconception care	33	..	..	..	..	..	..	..
predictive validity	33	..	..	..	..	..	..	..
predictors	46	..	..	..	..	..	18	..
prefrontal cortex	234	..	..	..	115	51	43	51
pregnancy	4316	842	1061	121	766	1026	775	988
pregnancy complication	1720	350	402	45	292	535	322	392
pregnancy diabetes mellitus	146	..	72	..	22	34	18	23
pregnancy disorder	52	..	21	..	..	..	..	..
pregnancy in adolescence	58	..	16	..	..	19	..	18
pregnancy outcome	621	126	177	..	83	151	125	140
pregnancy trimesters	35	..	..	..	..	..	..	..
pregnant woman	681	153	201	..	47	170	173	154
premature birth	579	111	192	..	82	130	112	114
prenatal	101	16	..	..	25	22	37	22
prenatal care	461	120	133	..	43	97	72	120
prenatal depression	34	..	20	..	..	..	..	..
prenatal development	48	..	..	..	17	..	..	..
prenatal diagnosis	64	..	..	..	..	19	15	..
prenatal drug exposure	1238	388	78	36	288	297	185	266
prenatal exposure delayed effects	1130	258	69	34	334	273	180	269
prenatal period	290	30	52	..	65	86	77	79
prenatal stress	146	..	..	..	43	45	44	38
prepulse inhibition	124	..	..	..	111	..	..	31
preschool	45	..	17	..	..	..	16	..
preschool child	2653	189	756	174	173	626	682	835
prescription	113	37	19	..	17	28	22	29
preterm birth	38	..	..	..	..	..	..	..
prevention	419	68	80	19	36	95	85	147
preventive medicine	36	..	..	..	..	..	..	..
primary care	44	..	..	..	..	..	..	17
primary medical care	130	..	35	..	16	40	34	46
primary school	65	..	26	..	..	..	..	20
primigravida	32	..	..	..	..	..	..	..
primipara	72	..	39	..	..	..	23	..
prison	37	..	..	..	..	..	..	..
prisoner	63	16	..	23	..	..	..	27
probability	78	19	..	..	..	24	..	21
problem behavior	122	..	32	..	..	31	33	45
problem solving	98	..	17	..	..	34	21	27
professional practice	56	..	15	..	..	..	..	17
professional-family relations	50	..	..	..	..	..	..	21
progesterone	39	..	18	..	..	..	..	..
prognosis	239	17	66	17	49	73	71	64
program development	42	..	..	..	..	..	..	..

program evaluation	74	..	24	..	..	..	..	26
prolactin	32	..	..	..	..	..	..	..
promoter region	88	..	..	..	28	32	..	32
protection	55	..	..	..	..	..	16	16
protective factors	45	..	..	..	..	..	..	..
protein blood level	77	..	..	..	40	..	..	20
protein expression	262	27	24	..	110	47	52	65
protein function	52	..	..	..	21	..	..	..
protein localization	30	..	..	..	..	..	..	..
psychiatric department	38	..	..	..	..	..	..	26
psychiatric disorder	30	..	..	..	..	..	..	25
psychiatric nursing	33	..	..	..	..	..	..	24
psychiatric treatment	64	..	..	..	18	18	20	36
psychoanalysis	115	..	20	22	25	22	28	24
psychoanalytic theory	36	..	..	..	..	..	..	..
psychodynamics	33	..	..	..	..	..	..	..
psychoeducation	89	..	17	..	21	26	44	31
psychological distress	35	..	..	..	..	..	17	..
psychological resilience	33	..	..	..	..	..	..	..
psychological theory	49	..	..	..	..	..	..	..
psychological well-being	52	..	..	..	..	..	24	..
psychologist	37	..	16	..	..	..	..	..
psychomotor performance	57	..	..	..	15	..	..	..
psychopathology	286	22	40	44	49	99	103	126
psychopathy	183	16	..	161	..	30	23	35
psychopharmacotherapy	75	..	19	..	19	40	26	23
psychosocial	31	..	..	..	..	..	..	..
psychosocial care	113	..	31	..	..	32	34	40
psychosocial disorder	103	..	17	..	..	16	30	47
psychosocial environment	38	..	..	..	..	..	..	..
psychosocial withdrawal	70	..	..	..	18	21	26	17
psychosomatic disorder	69	..	17	..	..	16	42	19
psychotherapy	459	23	127	59	62	156	134	122
psychotic disorders	762	45	41	59	762	216	115	204
psychotrauma	124	..	..	19	20	28	51	39
psychotropic drugs	170	20	33	..	46	67	57	74
puberty	83	..	29	..	..	15	..	..
public health	126	25	32	..	..	18	..	48
puerperal disorder	206	..	50	..	112	67	54	79
punishment	86	..	..	24	..	16	16	23
pyramidal nerve cell	34	..	..	..	18	..	..	..
quality of life	433	27	146	18	38	96	135	109
quetiapine	73	..	..	..	42	36	23	18
race	212	51	52	..	22	51	35	63
rat	749	109	75	..	249	136	131	180
reaction time	55	..	..	..	19	..	..	..
reading	30	..	..	..	..	..	..	..
real time polymerase chain reaction	73	..	..	..	22	20	..	21
rearing	100	15	..	..	17	22	30	18
recall	44	..	..	..	..	..	16	..

receiver operating characteristic	44	..	..	..	..	..	..	..
receptors, dopamine d2	38	..	..	..	..	..	..	..
receptors, glucocorticoid	38	..	..	..	..	16	15	..
receptors, n-methyl-d-aspartate	32	..	..	..	17	..	..	..
recognition	87	..	..	..	45	..	18	26
recreation	38	..	..	..	..	..	..	..
recurrence risk	30	..	..	..	..	..	..	..
recurrent disease	166	..	34	..	30	96	41	51
reference value	47	..	15	..	..	..	..	..
referral and consultation	104	..	27	..	..	29	26	35
reinforcement	90	28	19	..	..	..	24	..
reinforcement (psychology)	38	15	..	..	..	..	..	..
rejection (psychology)	49	..	..	..	..	21	..	..
relapse	60	15	..	..	24	18	..	..
relative	46	..	..	..	17	..	..	15
religion	102	29	..	..	17	22	25	21
remission	86	..	20	..	20	39	29	27
reproduction	61	..	17	..	..	..	..	..
reproductive health	38	..	..	..	..	..	..	..
residence characteristics	101	21	..	..	..	30	..	37
residential care	42	..	..	..	..	..	..	15
resilience	111	..	15	..	..	31	15	57
respiratory distress	30	..	..	..	..	..	..	..
respiratory tract disease	84	..	22	..	..	15	15	..
responsibility	45	..	..	..	..	..	..	..
restlessness	49	..	31	..	..	..	..	..
reverse transcription polymerase chain reaction	40	..	..	..	16	..	..	..
risk factor	4593	740	1000	396	758	1329	938	1395
risk reduction	111	29	22	..	19	24	22	27
risk-taking	108	55	..	..	..	..	..	29
risperidone	111	..	24	..	68	42	29	26
rural area	110	22	23	..	16	24	19	35
rural population	133	26	26	..	..	29	20	49
safety	48	..	18	..	..	..	..	..
saliva	97	..	..	..	..	39	43	24
saliva level	47	..	..	..	..	17	22	..
satisfaction	164	..	85	..	..	25	42	30
schizoaffective psychosis	85	..	..	..	85	50	15	33
schizoidism	32	..	..	19	18	..	..	..
schizophrenia	1566	48	42	96	1566	376	156	420
schizotypal personality disorder	51	..	..	51	38	..	..	16
school	238	44	32	..	16	43	73	90
school child	1553	156	358	99	169	390	498	497
school health service	30	..	..	..	..	..	..	..
seasonal variation	35	..	..	..	18	..	..	..
second trimester pregnancy	180	30	48	..	35	53	50	35
sedation	39	..	17	..	..	..	..	..
seizure	152	17	55	..	21	35	28	20
self assessment (psychology)	40	..	..	..	..	15	..	..
self care	59	..	34	..	..	..	..	..

self concept	551	42	202	55	40	140	163	128
self control	58	..	..	..	..	..	18	..
self disclosure	61	..	..	..	..	20	..	..
self efficacy	85	..	34	..	..	18	..	21
self esteem	225	27	82	19	15	62	74	57
self evaluation	77	..	21	..	..	21	21	16
self-esteem	31	..	..	..	..	..	..	..
self-injurious behavior	62	..	..	..	..	20	19	30
sensory gating	52	..	..	..	48	..	..	15
separation anxiety	267	..	29	18	27	69	195	75
sepsis	31	..	..	..	..	..	..	..
serotonin	539	33	93	..	75	281	166	139
serotonin 1a receptor	35	..	..	..	..	15	17	..
serotonin noradrenalin reuptake inhibitor	40	..	..	..	..	28	16	17
serotoninergic system	46	..	..	..	..	23	..	..
sertraline	156	..	41	..	25	95	57	33
sex characteristics	140	24	16	..	33	23	30	42
sex difference	1448	245	248	125	239	365	356	398
sexual abuse	239	40	40	48	30	83	80	93
sexual behavior	144	38	17	15	..	30	30	42
sexual crime	30	..	..	..	..	..	..	..
sexual development	47	..	..	..	..	..	..	18
sexual intercourse	35	..	..	..	..	..	..	..
sexual partners	34	..	..	..	..	..	..	..
sexuality	71	..	18	..	..	..	..	18
shame	53	..	17	..	..	..	..	..
short term memory	40	..	..	..	24	..	..	..
sibling	289	27	43	28	83	71	53	103
sibling relation	36	..	..	..	..	..	..	..
side effect	139	..	74	..	29	44	22	21
signal transduction	111	..	..	..	49	20	..	36
single parent	112	15	17	..	16	34	17	40
skill	41	..	..	..	..	..	..	16
sleep	492	..	441	..	..	45	41	36
sleep apnea	223	..	215	..	..	..	..	..
sleep deprivation	72	..	63	..	..	..	..	..
sleep disorder	997	21	997	..	21	73	93	60
sleep disorder assessment	30	..	30	..	..	..	..	..
sleep disordered breathing	252	..	252	..	..	..	..	..
sleep duration	230	..	222	..	..	18	..	..
sleep initiation and maintenance disorders	62	..	51	..	..	..	..	..
sleep parameters	37	..	37	..	..	..	..	..
sleep pattern	163	..	152	..	..	16	..	..
sleep problems	62	..	61	..	..	..	..	..
sleep quality	151	..	142	..	..	15	16	..
sleep stage	63	..	57	..	..	..	..	..
sleep wake disorders	140	..	139	..	..	..	..	..
sleep waking cycle	72	..	64	..	..	..	..	..
sleep-disordered breathing	46	..	46	..	..	..	..	..
smoking	956	367	167	61	93	174	135	241

smoking cessation	75	24	..	..	..	18	20	18
smoking habit	54	19	..	..	..	..	..	..
snoring	135	..	135	..	..	..	..	..
social adjustment	279	24	25	30	38	89	97	79
social anxiety	41	..	..	..	..	..	41	..
social aspect	287	62	62	19	44	49	61	84
social behavior	561	54	68	61	110	120	171	152
social behavior disorders	46	..	..	22	..	..	..	..
social care	32	..	..	..	..	..	..	..
social cognition	32	..	..	..	15	..	..	..
social competence	46	..	..	..	..	..	20	..
social disability	48	..	..	..	..	..	19	24
social environment	436	77	60	87	57	125	74	132
social evolution	36	..	..	..	..	..	..	..
social interaction	378	22	60	22	94	83	134	95
social isolation	168	..	24	16	46	44	42	56
social network	30	..	..	..	..	..	..	..
social perception	91	..	15	..	..	23	22	22
social phobia	311	25	28	23	22	83	312	86
social problem	118	18	24	..	..	29	38	41
social psychology	379	57	76	42	40	105	97	122
social stigma	57	..	..	..	..	16	..	25
social support	917	75	281	48	85	240	198	269
social welfare	35	..	..	..	..	..	..	..
social work	72	19	..	..	..	16	..	31
socialization	105	..	..	23	..	28	28	21
socioeconomics	1854	310	380	134	271	427	345	642
sodium chloride	62	18	..	..	24	..	..	..
somatoform disorder	202	..	27	28	36	47	188	51
somnolence	75	..	65	..	..	..	..	..
spatial memory	45	..	..	..	21	..	..	15
speech	42	..	..	..	..	..	..	..
speech disorder	52	..	24	..	..	..	..	..
spontaneous abortion	125	16	42	..	26	43	36	19
spouse	75	..	18	..	..	27	15	21
startle reflex	101	..	..	..	71	..	..	22
stereotypy	44	..	..	..	..	..	16	..
stigma	75	..	..	..	..	..	..	32
stillbirth	97	24	25	..	18	22	..	27
stimulus response	36	..	..	..	..	..	..	..
street drug	50	38	..	..	..	..	..	..
stress	1897	118	384	79	195	539	592	577
stress, physiological	60	..	..	..	17	19	19	20
student	318	101	60	20	..	46	88	71
substance abuse	792	385	77	106	117	230	179	288
substance use	181	97	..	16	24	37	31	53
substance use disorder	713	386	32	82	68	173	110	233
sucrose	39	..	..	..	..	..	..	..
suicidal ideation	299	43	49	33	49	143	86	115
suicide	302	35	40	38	74	126	58	149

suicide attempt	282	56	29	45	52	141	80	125
survival	48	..	..	..	..	..	..	16
survival rate	32	..	..	..	..	..	..	..
survivor	54	..	..	..	..	17	24	..
swimming	149	..	20	..	16	65	33	41
symptom assessment	45	..	16	..	..	..	15	..
synapse	37	..	..	..	24	..	..	..
syndrome	42	..	..	..	..	..	..	16
systolic blood pressure	36	..	17	..	..	..	..	..
task performance	184	18	..	..	62	44	49	32
teacher	119	..	15	..	..	15	27	62
television	30	..	..	..	..	..	..	..
temperament	346	19	96	53	..	93	124	58
temporal lobe	40	..	..	..	23	..	..	..
teratogenicity	60	..	..	..	..	29	..	..
testosterone	36	..	..	..	..	..	..	..
thalamus	31	..	..	..	20	..	..	..
theory of mind	33	..	..	..	..	..	..	..
therapy effect	65	..	17	..	..	20	24	..
third trimester pregnancy	347	43	121	..	34	98	91	64
threat	33	..	..	..	..	..	23	..
thyroid disease	30	..	..	..	..	..	..	..
tic	53	..	..	..	..	..	41	16
tobacco	134	92	..	..	..	17	..	21
tobacco dependence	141	89	..	..	..	33	30	48
tobacco smoke pollution	34	..	15	..	..	..	..	..
tobacco use	59	30	..	..	..	..	..	..
tobacco use disorder	63	35	..	..	..	15	..	22
toddler	51	..	23	..	..	..	..	..
tonsillectomy	37	..	35	..	..	..	..	..
topiramate	32	..	..	..	..	16	..	..
traffic accident	39	..	..	..	..	..	..	..
training	47	..	..	..	..	..	..	21
trauma	97	..	..	..	18	23	29	35
treatment	112	..	30	..	..	28	44	22
treatment duration	161	33	48	..	20	37	42	27
treatment indication	35	..	18	..	..	..	..	..
treatment outcome	777	90	233	41	79	199	242	147
treatment planning	50	..	15	..	..	15	..	22
treatment refusal	36	..	..	..	..	..	..	..
treatment response	141	..	37	..	18	57	55	22
tremor	51	..	18	..	..	22	..	..
tricyclic antidepressant agent	70	..	21	..	..	40	19	21
trust	33	..	..	..	..	..	..	..
tumor necrosis factor	112	..	..	..	66	20	..	18
twins	234	55	35	33	31	58	70	61
umbilical cord blood	62	..	..	..	..	27	..	..
unclassified drug	252	29	47	..	88	53	32	64
underweight	45	..	30	..	..	..	..	..
uniparental disomy	36	..	16	..	15	..	..	..

university	83	30	15	..	..	..	20	22
university hospital	69	..	20	..	..	15	19	17
unplanned pregnancy	66	..	29	..	..	16	..	17
unspecified side effect	35	..	..	..	..	..	..	..
unwanted pregnancy	48	..	..	..	..	..	..	17
upregulation	54	..	..	..	27	..	..	17
urinalysis	62	39	..	..	..	..	..	..
urinary tract infection	39	..	19	..	..	..	..	..
utilization	59	..	..	..	..	..	..	32
vaccination	33	..	18	..	..	..	..	..
valproic acid	107	..	29	..	36	60	24	27
vasopressin	33	..	..	..	..	..	..	..
venlafaxine	100	..	27	..	16	69	36	22
verbal behavior	56	..	..	..	..	..	..	19
verbal memory	36	..	..	..	24	..	..	..
vertical transmission	30	..	..	..	..	..	..	..
very elderly	53	..	..	..	..	22	..	18
very low birth weight	33	..	..	..	..	..	..	..
victim	77	22	..	15	..	20	18	22
videotape recording	38	..	..	..	..	..	..	..
violence	277	73	32	51	42	57	48	100
virus infection	36	..	..	..	23	..	..	..
vitamin d	62	..	20	..	33	..	..	..
vitamin d deficiency	36	..	..	..	24	..	..	..
vitamin supplementation	44	..	19	..	..	..	..	..
vocalization	56	..	..	..	..	..	19	..
vomiting	151	..	119	..	..	..	..	..
vulnerability	39	..	..	..	..	17	..	..
vulnerable population	80	..	..	..	..	32	..	23
wakefulness	123	..	108	..	..	..	..	..
war	57	..	..	..	..	..	17	28
weaning	71	..	19	..	15	..	..	15
weight gain	217	17	127	..	30	37	38	47
weight reduction	114	..	87	..	..	..	..	15
wellbeing	281	29	74	..	..	61	76	88
western blotting	56	..	..	..	24	..	..	15
wheezing	38	..	28	..	..	..	..	..
white matter	54	..	..	..	27	..	..	..
withdrawal syndrome	183	129	21	..	15	19	26	16
women	100	25	33	..	..	31	18	21
women’s health	155	17	60	..	16	44	26	34
working memory	82	..	..	..	53	..	..	24
wounds and injuries	47	..	..	..	..	..	..	16
young adult	2751	481	629	210	417	741	610	884
